# Fatigue Crack Growth in Rubber: Comparability of Test Results, Material Effects and Transfer to Component Life—A Critical Review

**DOI:** 10.3390/polym18202476

**Published:** 2026-10-10

**Authors:** Jeong-Hwan Yoon, Kyoung-Ho Shin, Ye-Won Park, Yeon-Jee Cho

**Affiliations:** Sustainable Materials R&D Center, Korea Automotive Technology Institute, 303 Pungse-ro, Pungse-myeon, Dongnam-gu, Cheonan-si 31214, Chungnam, Republic of Korea

**Keywords:** rubber, data comparability, fatigue crack growth, tearing energy, test methods, strain-induced crystallisation, filler, component fatigue life

## Abstract

Fatigue crack growth (FCG) relations rank rubber compounds and predict component life, yet a reported rate–tearing-energy curve depends on how it was measured as well as on the compound. This critical review treats the test variables as criteria for deciding whether two results may be compared. Specimen geometry, energy accounting, load minimum, frequency, waveform, specimen temperature, crack preparation, imaging, rate extraction and data independence shape the measured response. A two-stage, seven-item checklist and an eight-domain minimum reporting set are proposed, and the evidence on backbone, filler, cure system, temperature, ageing and chemical environment, graded by strength, is read against them. Formulation rankings hold only within matched test conditions: when compounds differ in exponent, the power-law curves cross, and rankings can reverse within or beyond the tested energy range. Where exponents were reported for both, natural rubber mostly showed a lower exponent than styrene–butadiene rubber, but the backbone was not isolated and exponent values differ between programmes. Component transfer requires geometry-specific crack-driving histories, defect criteria, thermal and load histories, and independent validation. Quantitative comparison needs a shared endpoint and energy convention, with other items matched or bounded by measurement; a single-compound inter-laboratory round-robin would test the checklist.

## 1. Introduction

Rubber components sustain large, repeated deformations while defects, hysteretic heating, environmental exposure and contact damage evolve together. Tyres, mounts, isolators, seals and track components therefore pose a coupled durability problem, in which the material has to resist both the appearance of damage and the growth of cracks under changing mechanical and thermal conditions [1,2,3,4]. A fatigue life measured on an uncracked specimen and a crack growth rate measured on a pre-cracked one answer different questions, and treating them as one property obscures the assumptions needed to rank formulations or predict service life.

Tearing-energy-based fatigue crack growth (FCG) describes crack propagation in a form that can be integrated into a life. A reported relation between crack growth rate, d*c*/d*N*, and tearing energy, *T*, nevertheless reflects the test as well as the composition. Specimen geometry and constraint, the stored-energy convention, the load minimum, waveform and frequency, crack preparation, temperature, imaging and rate extraction all shape the apparent response. Two coefficients with the same units can therefore be incomparable, while differently configured experiments may still support a controlled qualitative contrast.

Among the broad reviews of rubber fatigue, Mars and Fatemi separated crack nucleation and crack growth approaches and connected fracture mechanics to life prediction [1], and Tee et al. updated that survey [2]. Gehling et al. gave the closest earlier FCG-centred treatment among the reviews examined here [3], and Wang et al. reviewed tearing-energy calculations, specimen geometries and test approaches extensively, together with models and applications [4]. Qiu et al. explained specimen classes and propagation laws and reviewed mechanical, thermal and environmental factors in fatigue life [5]. Głowacka et al. reviewed the fatigue lifetime of rubber composites, mainly with rubber as the matrix, and identified manufacturing technology as the primary influence and the matrix–reinforcement interface as also significant [6]. Application-focused reviews address tyre modelling [7], and Tsunoda traced the lineage of methods from energy-balance growth measurements to crack tip analyses [8]. The closest of these reviews [2,3,4] recognise that specimen choice and test variables matter, but they organise the field by analysis method, factor, material family or application and treat measurement variables as influencing factors; Table 1 compares seven representative reviews with the present one. In this review, they are instead treated as criteria for deciding whether results may be combined, and the question of whether two reported results can be placed side by side is examined before any synthesis.

Three recent reviews that compile thresholds across polymer classes show where that question sits. Baur and Amstad asked whether elastomers can combine stiffness, toughness and fatigue resistance, setting out geometry requirements and a rate-extraction convention for a fatigue threshold [10]. Ning et al. surveyed fatigue mechanisms, mechanics and anti-fatigue design across thermosets, thermoplastics, elastomers and hydrogels [9], and Steck et al. organised threshold behaviour in polymeric materials around the deconcentration of stress at the crack tip [11]. Each assembles thresholds from many separate reports on one Ashby-type plot without recording the load ratio, frequency, thermal state, crack resolution or replicate hierarchy of each value. One such plot draws on twenty-three cited sources across four polymer classes [9], and another includes filled elastomers, natural and butadiene rubbers, poly(dimethylsiloxane) (PDMS) and poly(methyl methacrylate) (PMMA) [10]. Steck et al. made the difficulty explicit, noting that limited crack resolution inflates an apparent threshold, that reported resolutions vary over about three orders of magnitude and that “one should be careful in comparing thresholds reported in different papers” [11].

Building on that caution, the aim of this review is to establish when two FCG results may be compared quantitatively and to read the evidence on material, thermal and environmental effects against that criterion. To this end, a two-stage, seven-item checklist is proposed, in which an endpoint check precedes six compatibility checks on geometry and constraint, energy definition, load minimum, rate and history, thermal state and data reduction. A minimum reporting set in eight domains complements it. Read against the checklist, formulation rankings hold only within matched test conditions and can reverse even within the tested energy range. Even the lower exponent of strain-crystallising natural rubber relative to non-crystallising styrene–butadiene rubber is an ordering seen in most exponents reported for both, none from a study that isolated the backbone. Transfer to a component further requires a geometry-specific crack-driving history, defect criteria and operative thermal and load histories, which coupon data do not supply. Since FCG relations serve both compound ranking and life prediction, these conditions decide which published relations can inform either use.

## 2. Literature Search and Selection

**Figure 1:**
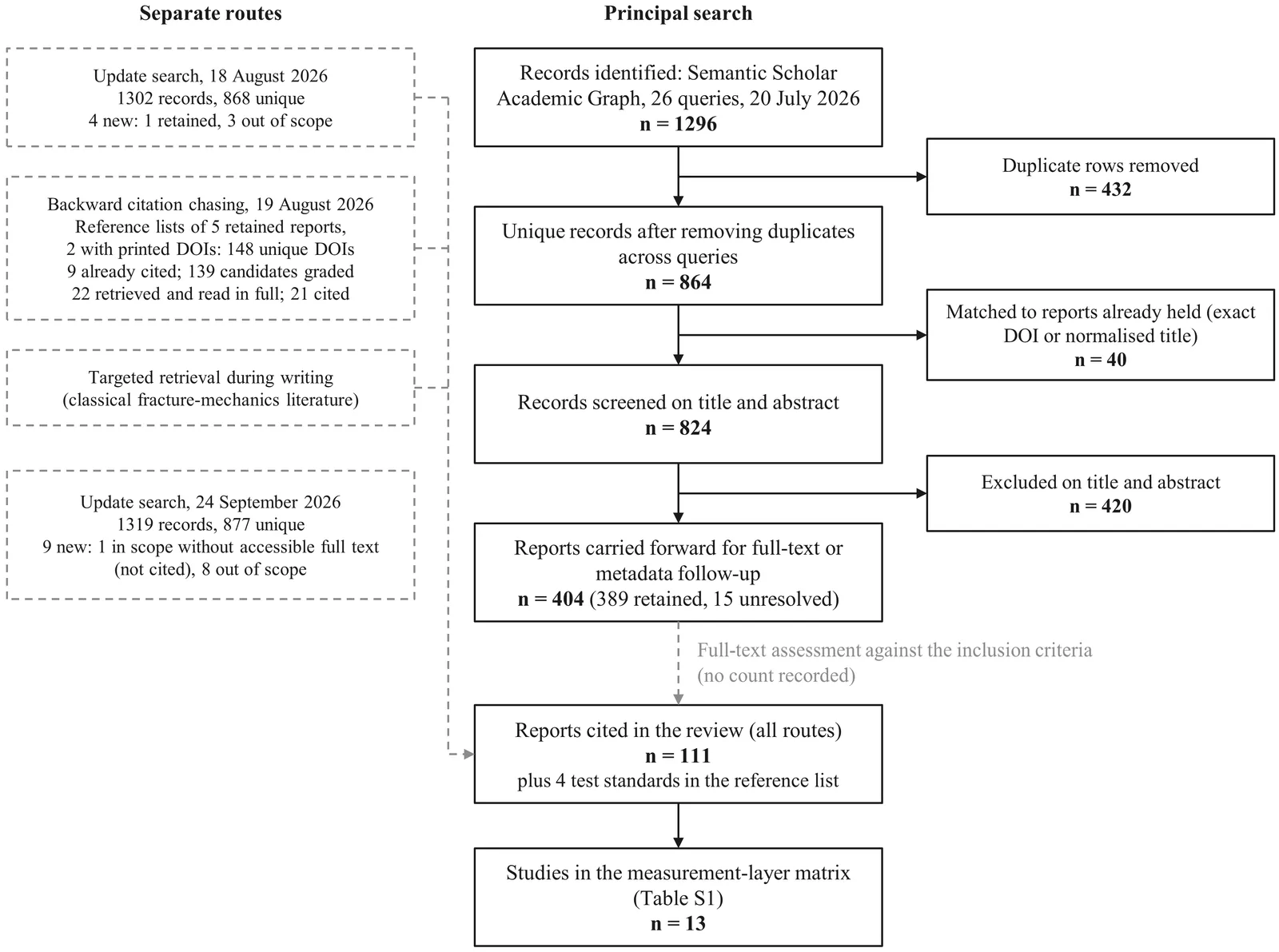
Identification, screening and inclusion of the literature reviewed; flow diagram drawn by the authors. The principal search comprised twenty-six archived Semantic Scholar queries of 20 July 2026; forty unique records that matched reports already held, by exact DOI or normalised title, were handled through the existing record before title-and-abstract screening. The two update searches, backward citation chasing and targeted retrieval during writing are separate routes and are not counted in the principal-search flow; the second update search added no cited report. Because the route by which each cited report entered the review was not recorded for every report, dashed links carry no count, no full-text exclusion count is given and the cited set is not divided by route. The reference list, of 115 entries including four test standards, is the record of inclusion; query strings, yields and stage records are given in Appendix S1.

This is a critical review based on a documented literature search. It is not systematic or exhaustive. The Semantic Scholar Academic Graph was searched on 20 July 2026 without a lower publication-date limit, and the search was updated on 18 August and 24 September 2026. This database was chosen because it covers materials, mechanics and polymer journals and because its queries can be archived with their yields and re-run (Appendix S1). Targeted retrieval from reference lists and the classical fracture-mechanics literature supplemented the search. The twenty-six query exports of 20 July contained 1296 records, 864 of them unique; forty matched reports already held, and the remaining 824 were screened on their titles and abstracts, and 404 were carried forward for full-text or metadata follow-up (Figure 1). Records outside the database’s index, such as some technical reports, theses and non-English papers, may have been missed, and because each query carried one term per concept, terms such as ozone, oxidation, fluid, oil and water were not sent (Appendix S1); backward citation chasing and targeted retrieval reached reports outside the queries. The principal conclusions rest on within-study contrasts and recorded test conditions rather than on how often a finding recurs, and a statement that something was not found refers to the literature reviewed here. Each source used for a quantitative statement was checked against its text, and an inaccessible or internally inconsistent source supports only what its accessible text states.

A study was considered when it addressed rubber or a relevant elastomer-containing system and reported direct pre-cracked FCG, an explicit bridge to another fatigue or component endpoint, a mechanism or method needed to interpret FCG, or foundational context. Quantitative use of a result required a primary full text in which the items of Section 3.5 could be assessed, and an unreported item was treated as unresolved. Reviews and foundational papers provide context but do not replace primary quantitative evidence. Only rates stated in the text or tables of the original papers are quoted because a rate read off a plotted curve adds digitisation uncertainty.

Where the number of independent specimens behind a rate curve is not stated, the study is retained without conversion to specimen-level statistics. Related publications that share specimens, crack histories, coefficients or validation data are treated as one dataset family (Section 4.7). Where the available texts leave the relation between two studies unresolved, the two are not counted as independent. No formal study-level risk-of-bias score was assigned because the appraisal concerns the comparability of results; the synthesis is narrative.

## 3. Fracture Mechanics Basis

### 3.1. Tearing Energy

Rubber fracture is best approached through an energy balance instead of a local stress criterion alone. Griffith established that crack extension becomes energetically admissible when the reduction in potential energy compensates for the energy required to create new surfaces [12]. His energy balance carries over to elastomers, although his linear-elastic, brittle-solid assumptions do not.

Rivlin and Thomas adapted this reasoning to large elastic deformation in rubber sheets by defining a characteristic tearing energy. For a crack of length *c* in a sheet held at fixed boundary displacement *l*,

(1) $$T=-\frac{1}{t}{\left(\frac{\partial U}{\partial c}\right)}_{l}=-{\left(\frac{\partial U}{\partial A_{\mathrm{cr}}}\right)}_{l},$$

where *U* is the stored elastic energy, *t* the undeformed sheet thickness and *A*_cr_ = *tc* the conventional projected crack area [13]. The tearing energy *T* is the mechanical energy available per unit projected crack area under the prescribed boundary condition, and it was not identified with a thermodynamic surface free energy.

Evaluating *T* is geometry-dependent. In the valid central region of a sufficiently long pure-shear strip, the definition reduces to *T* = *Wh*, with *W* the imposed strain-energy density and *h* the undeformed height [13]. Greensmith derived an energy-supply expression for a small edge cut in simple extension through a geometry function *K*(*λ*) [14]. A correctly evaluated *T* should not depend on the specimen, and Rivlin and Thomas established the quantity as characteristic by showing that trouser, pure-shear and simple-extension specimens of the same vulcanisates gave consistent values [13]. The strain concentration at the tip, in contrast, depends on tip shape and is difficult to measure. Thomas measured it around a round-ended incision and recovered the tearing energy from it independently, the two estimates agreeing within 5–10% in simple extension and about 5% in pure shear at strains below 200% [15]. The global definition holds whatever the incision shape, although for round-ended incisions, the tearing energy at rupture scaled with tip diameter [15] (Section 4.5). Disagreement between geometries calls for examining the energy convention, dissipation, rate and material state before the property is judged geometry-dependent.

A reported critical tearing energy thus marks a transition for a particular material state and protocol, and for cyclic data, the energy measure and cycle definition also have to be stated. Throughout, *c* denotes crack length in coupon tests and *a* crack size in life integrations, where the growth law d*c*/d*N* is evaluated at a geometry-specific *T*(*a*).

### 3.2. Crack Growth Regimes and the Power Law

**Figure 2:**
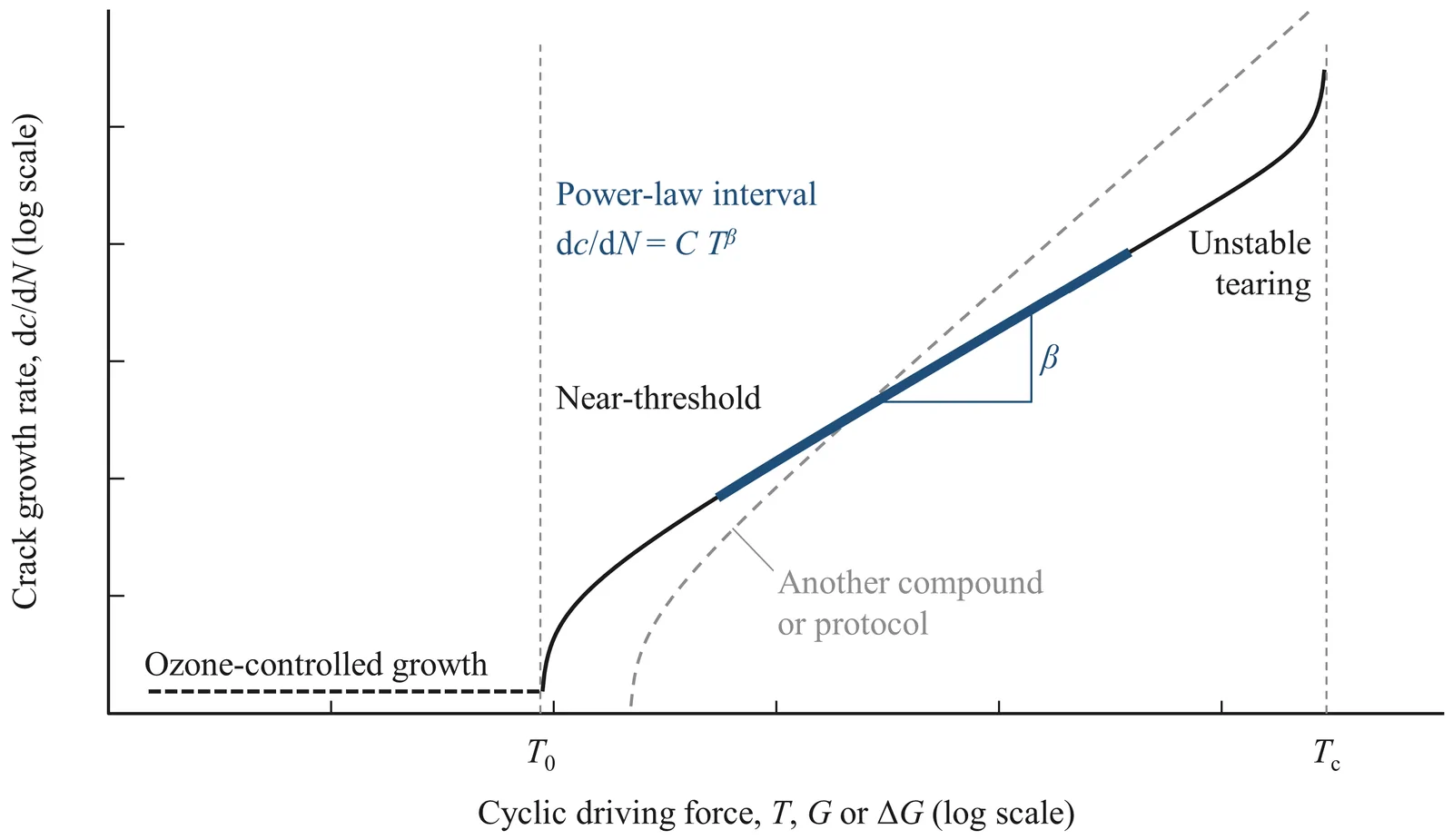
Crack growth curve on logarithmic axes; schematic drawn by the authors. The horizontal coordinate is a source-defined cyclic driving force such as *T*, *G* or Δ*G*. Below the threshold tearing energy *T*_0_, the broken line marks ozone-controlled growth, as Lake and Lindley described for particular vulcanisates in air [16]; above it, the rate rises through a near-threshold region into a power-law interval, d*c*/d*N* = *C* *T^β^*, whose exponent *β* is the slope on these axes, and turns upwards into unstable tearing at the critical tearing energy *T*_c_. The grey dashed curve is another compound or protocol. The curve is conceptual and does not reproduce a source dataset.

Rubber crack growth curves can display several kinetic regions, from very low or undetected growth through stable slow growth and an intermediate power-law-like interval to rapid growth towards unstable tearing (Figure 2). Regions may be absent or merge. Environmental mechanisms form a separate axis, since ozone-dominated and mechanico-oxidative growth can occupy different parts of a low-energy response [16].

The classical power laws were each established for a particular rubber under particular test conditions. For gum natural rubber (NR) under low-frequency relaxed cycling, Gent, Lindley and Thomas found an approximately quadratic dependence of cut growth per cycle on maximum tearing energy [17], and earlier, Thomas had found analogous *T*^2^ relations for static and cyclic cut growth in the same material [18]. In non-strain-crystallising styrene–butadiene rubber (SBR), in contrast, Lake and Lindley found approximately fourth-power behaviour over the applicable interval, followed by a high-*T* departure before catastrophic tearing [19]. The relation

(2) $$\frac{dc}{dN}=CT^{\beta }$$

is therefore a fitted descriptor over a given interval. The exponent *β* depends on the material and the test conditions, and the coefficient *C* additionally depends on the units assigned to *T* and d*c*/d*N*. Neither should be averaged across studies before the alignment set out in the comparison checklist. A slope change signals a transition or a change in effective description and does not justify one broad fit.

Because the exponent is dimensionless, it can be compared where the coefficient cannot, provided the checklist items are aligned. The classical *T*^2^ and *T*^4^ values are nevertheless not constants of either polymer, as the comparison checklist shows below when applied to the ordering between strain-crystallising and non-crystallising rubber.

Modern evidence reinforces the same point. In NR/butadiene rubber (BR) tread blends, Ghosh et al. observed bilinear behaviour and composition-dependent transitions at roughly 20–35% strain. Higher-BR blends ranked better below the transition and the ranking reversed above it, while temperature, load ratio and waveform also affected the response [20]. Duncan et al.’s continuous multi-region formulation is likewise an organising model whose parameters are specific to the non-strain-crystallising rubbers examined [21].

### 3.3. Meanings of “Threshold”

Lake and Lindley identified, for the vulcanisates and atmosphere examined, a level near 0.05 kgf cm^−1^ (approximately 49 J m^−2^) at the onset of mechanico-oxidative growth, below which residual growth was attributed mainly to ozone. The rate increased by at least about two orders of magnitude across the narrow transition [16]. Because this level is specific to those compounds and that environment, it cannot be read as a universal fatigue threshold.

In the literature reviewed here, the word “threshold” covers at least seven quantities that do not convert into one another:

1. An operational non-detection or run-out level for a pre-cracked specimen, fixed by protocol, resolution and cycle window;
2. A mechanism- or regime-transition energy, such as the one just described;
3. The threshold tearing energy of a multi-regime fit;
4. An unnotched fatigue limit or run-out criterion;
5. A near-equilibrium threshold tear strength measured on swollen specimens at a low tearing rate;
6. A model-defined intrinsic strength and its estimation procedure;
7. An ozone threshold on a prestrained specimen without cycling.

Because each is defined by its own procedure, a value carried from one to another is a substitution rather than a conversion.

### 3.4. Endpoints and Intrinsic Resistance

Overlapping terminology can make distinct fatigue questions appear to measure one property. Table 2 separates experimental and life endpoints from energy-accounting quantities. The two cyclic endpoints most easily confused are detection-defined crack initiation, which counts cycles to a defined observable event in an initially uncracked specimen, and pre-cracked FCG, which measures the advance of an existing crack against a chosen measure of driving force.

**Table 2:** Fatigue and fracture endpoints and energy-accounting quantities. Endpoint rows list experimental and life endpoints; energy-quantity rows list accounting quantities that may be applied within several endpoints.

Quantity	Starting State, Input and Observable	Design Question	Not Directly Poolable with
*Endpoints*			
Monotonic crack onset	Notched/pre-cracked body, rising load/deformation; force and deformation at declared onset give effective resistance	When does propagation begin at the stated geometry and rate?	Controlled steady tearing, cyclic growth or life without an explicit bridge
Controlled steady tearing	Existing crack, controlled monotonic or steady propagation; steady force, crack speed or speed-dependent work of fracture	What resistance accompanies steady propagation in the stated state?	Monotonic onset or cyclic d*c*/d*N*; speed and energy convention remain part of the result
Operational pre-cracked FCG non-detection or run-out threshold	Existing crack, declared low-driver cyclic window; detectable advance or run-out at stated resolution and cycle count	Below what protocol-defined driver was growth not detected?	Mechanism- or regime-transition energy, unnotched fatigue limit, or proof of zero physical growth
Mechanism- or regime-transition energy	Existing crack across a range spanning a response change; change in attributed mechanism, fitted slope or kinetic regime	Where does response change for stated material, environment and protocol?	Non-detection threshold, unnotched fatigue limit, or another compound’s transition value
Detection-defined crack initiation	Uncracked or nominally smooth specimen, cyclic loading; first crack meeting a declared detection criterion	When is a technically detectable crack first observed?	Physical nucleation time or pre-cracked propagation life; detection can follow nucleation
Pre-cracked FCG	Known starter crack, specified cyclic energy or load history; d*c*/d*N* against *T*, *G*, Δ*G* or another declared driver	How fast does an existing crack advance under the stated conditions?	Initiation, total life, wear, or coefficients from incompatible test conditions
Crack-to-failure integration	Calibrated FCG law, *a*_0_, *a*_f_, geometry-dependent *T*(*a*), stated update rules; propagation life $N_{p}=\int_{a_{0}}^{a_{f}}[dc/dN{]}_{T(a)}^{-1}\;da$; *a*_0_, *a*_f_ uncertainty propagates	What propagation life follows from a given initial crack and driver history?	Measured total life unless initiation and transfer assumptions are validated
Unnotched fatigue limit or run-out criterion	Uncracked specimen, specified cyclic loading level; survival/non-failure over declared cycle count and failure definition	What operational loading level meets the specified run-out criterion?	Pre-cracked FCG threshold, mechanistic nucleation threshold, or infinite-life claim
Complete-fracture fatigue life	Specimen cycled to a declared macroscopic failure criterion; *N*_f_, S–N or ε–N relation, or survival distribution	How long does the tested specimen survive to the declared event?	Pre-cracked d*c*/d*N*; *N*_f_ may combine initiation, distributed damage, coalescence and propagation
Dissipated-energy life model	Uncracked body, specified cyclic history; life related to continuum dissipation at a critical location, no crack tracked	What life follows from the dissipation field of an uncracked body?	Detection-defined initiation and pre-cracked growth; endpoint normally complete fracture, not nucleation
Wear, cut-and-chip or repeated-impact material loss	Surface under friction, cutting, impact or abrasive cycles; material loss, chipping, recession or damage score	How does the surface resist removal under the stated contact system?	FCG rate or tearing-energy ranking, without a validated transfer relation
*Energy quantities*			
Model-defined local crack tip fracture energy	Adopted local crack-tip or surface-creation model; energy assigned to locally creating projected crack area	What local energy scale is required within the adopted model?	Macroscopic work of fracture; “intrinsic” requires a stated estimation procedure
Measured macroscopic fracture resistance	Whole specimen, resolved tip and surrounding dissipative fields; external work or stored-energy release per projected crack area	What resistance does the material–protocol system exhibit?	Model-defined local energy, or another protocol’s value without an equivalence argument

Threshold tear strength shows how closely an endpoint is tied to its procedure. Gent and Tobias measured a near-equilibrium threshold tear strength at low tearing rates, high temperatures and high swelling and obtained the predicted square-root scaling with network-strand molecular weight. Their quantity is a tear-strength endpoint, distinct from a cyclic d*c*/d*N* threshold [22]. Ahagon and Gent had measured 40–80 J m^−2^ earlier for two polybutadiene elastomers torn slowly, hot and swollen. The value was independent of temperature and tearing rate, and of the degree of swelling and the swelling liquid, provided a correction is made for the reduced areal concentration of chains, but it fell as crosslinking rose [23].

Life predictions add further endpoints (Table 2). Crack-to-failure integration predicts propagation life from an initial crack size *a*_0_ to a terminal size *a*_f_, whereas complete-fracture life can also contain initiation, distributed damage, coalescence and final propagation. Agreement between the two therefore has to be shown by validation and does not follow from the definitions. Interpreting strain–life behaviour through growth from small pre-existing flaws provided an early bridge [17] but did not remove flaw-size uncertainty. Wear and cut-and-chip damage form another family governed by contact and evolving surface geometry, and their rankings cannot be relabelled as d*c*/d*N*–*T* rankings without a validated transfer relation.

The energy quantities in Table 2 need a similar separation because the model-defined energy assigned to local surface creation differs from the macroscopic work measured in a fracture experiment, which can include bulk viscoelastic dissipation and other process-zone contributions. Kroon’s finite-strain steady-crack model separated local crack tip surface energy from total energy release, and within its calibrated conditions, the gap was governed mainly by bulk viscoelastic dissipation, with inertia comparatively small over the lower modelled crack-speed range [24]. The term “intrinsic” therefore has to identify both the idealised contribution and the procedure used to estimate it, and a critical tearing energy, power-law coefficient or d*c*/d*N* value is a material response only within a controlled state and protocol.

### 3.5. A Two-Stage Comparison Checklist

The endpoint distinctions above and the test variables examined below support a sequential comparison in two stages, seven items in all (Table 3). A single endpoint check decides whether two results answer the same experimental question, and six compatibility checks decide whether results that do were obtained in compatible states. Both stages should be passed, in order, before two numerical FCG relations are placed on the same plot or entered into one regression.

**Table 3:** Two-stage comparison checklist for pre-cracked FCG results, with the evidence basis of each item and the consequence when it is unresolved. Item 1 is the first stage and items 2–7 the second. A within-study experimental contrast is a measurement of the relation within one programme when only that item was varied, or re-evaluated on the same tests, whether or not the relation changed. A methodological or mechanistic basis is one for which the reviewed studies provide no such contrast. A bounded comparison also requires the contrast to have been measured on the same base polymer as the results compared.

Item	What Must Match	Evidence Basis	Consequence If Not Matched
1. Endpoint	Both results measure pre-cracked crack growth per cycle rather than initiation life, complete fracture, wear or cut-and-chip damage (Table 2)	Methodological: the endpoints measure different quantities	Gatekeeping: no quantitative comparison
2. Geometry and constraint	Specimen configuration, admissible crack-length range and intended plane-stress or plane-strain state (Section 4.1, Section 4.2 and Section 4.3)	Within-study contrast for pure-shear proportions: width-to-height ratio and notch configuration left the rate–energy relation of a filled NBR unchanged over the tested range [25]. Methodological across geometries: the DCB–SEN contrast also changed frequency and driving-force calculation [26]	Bounded comparison within the tested pure-shear proportions; otherwise, qualitative or within-study
3. Energy definition	*T*, *G* or Δ*G* from equivalent quantities and the same loading or unloading convention (Section 4.4)	Within-study contrast: loading- and unloading-branch values from the same tests differ by a factor fixed by the compound’s hysteresis ratio [27]	Gatekeeping for coefficients, which stay paired with their energy definition; exponents across branches only where both branches or the hysteresis ratio are reported for the same tests
4. Load minimum	Force, displacement, strain or tearing-energy ratio defined consistently, including any positive minimum load (Section 5.1)	Within-study contrasts: positive versus negative load ratios [28]; displacement ratios above 0.23 lowered the rate below the *R* = 0 power law in cyclic *J*-integral range in NR [29]; no marked load-ratio effect in a filled NBR [25]	Bounded comparison inside the measured ratio range; otherwise, qualitative or within-study
5. Rate and history	Frequency, waveform, dwell, preconditioning and constant- or variable-amplitude history (Section 4.5, Section 5.2 and Section 5.4)	Within-study contrasts: frequency [30]; dwell-containing versus sinusoidal waveforms [28]; pulse versus sine excitation [31]; increasing versus decreasing amplitude histories, frequency, and waveform (no marked effect) in a filled NBR [25]	Bounded comparison inside the measured range of the variable; otherwise, qualitative or within-study
6. Thermal state	Chamber temperature, specimen temperature, prior ageing and self-heating distinguished (Section 5.3 and Section 8)	Within-study contrasts: test temperature, with the magnitude stated only approximately because the prefactor is not tabulated [32]; prior thermal ageing [33]. Mechanistic for heat build-up (measured as substantial at large loads and high frequencies [28]) and for the chamber–specimen gap, which none of the FCG tests in Table S1 recorded	Bounded comparison inside the measured range of prior ageing, or of test temperature where the within-study contrast tabulates the fitted coefficients that carry its magnitude; the chamber–specimen gap is resolved only by measured specimen temperature; otherwise, qualitative or within-study
7. Data reduction	Crack resolution, replicate hierarchy, transient exclusion and rate-fitting procedure (Section 4.6 and Section 5.5)	Within-study contrast: local and global differentiation of one crack record [34,35]. Methodological for resolution: too coarse a resolution overestimates a threshold [11]	Comparison after re-deriving rates from the raw crack records under one reduction procedure, with crack resolution and replicate hierarchy matched as recorded; otherwise, qualitative or within-study

The endpoint is the only full gate because results with different endpoints measure different quantities. The energy definition is a gate for the coefficient, which stays paired with its energy definition; an exponent can be compared across energy branches only where both branches, or the hysteresis ratio linking them, are reported for the same tests [27,36]. For geometry, load minimum, rate and history, and thermal state, partial compatibility supports a bounded quantitative comparison only where a within-study contrast has measured the effect of that item over the relevant range on the same base polymer, and the comparison must then state that bound. Otherwise, an unresolved item limits the result to qualitative or within-study synthesis and excludes its coefficient from a cross-study quantitative model. Data reduction is matched instead: fitting and transient exclusion by re-deriving rates from the raw crack records under one procedure; crack resolution and replicate hierarchy as recorded. If laboratories testing one compound batch in one geometry still disagree beyond their within-laboratory scatter after every item has been aligned, the residual points to a state variable the checklist does not yet name. A round-robin of the kind proposed in Section 10, with one geometry repeated across laboratories, would therefore test the completeness of the checklist.

## 4. Test Methods: Specimens, Energy Accounting and Crack Observation

A fatigue crack growth curve is readily taken as a fingerprint of a rubber compound. In practice, the relation between crack growth rate and tearing energy is filtered through a measurement layer, which comprises geometry and constraint, the definition of the driving force, crack tip preparation and conditioning, and the procedure that converts images of a moving crack into a rate.

Table S1 sets geometry, energy convention, load minimum, frequency and thermal state beside material identity for thirteen FCG studies selected to span the contrasts drawn on below.

### 4.1. Pure-Shear Specimens

**Figure 3:**
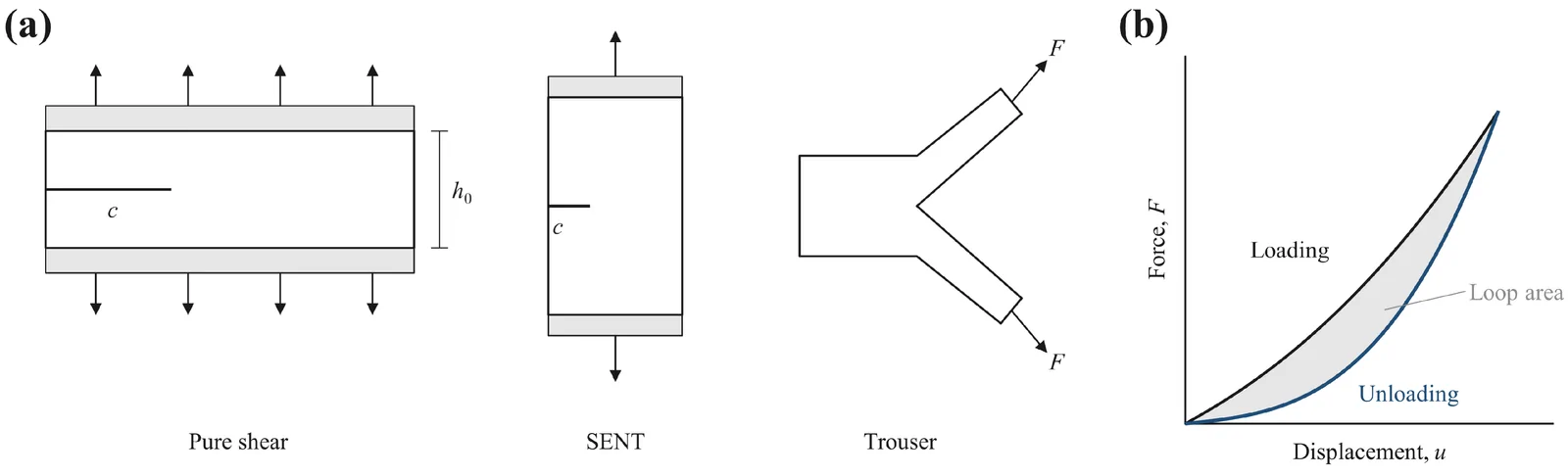
Specimen geometries and energy branches; schematic drawn by the authors. (**a**) Pure-shear, single-edge-notched tension (SENT) and trouser specimens; *c* is the crack length, *h*_0_ the undeformed height of the pure-shear strip between the grips (written *h* in Equation (3)) and *F* the force on each trouser leg, and arrows show the loading direction. (**b**) Force *F* against displacement *u* over one cycle, with the loading and unloading branches and the shaded loop area between them.

Pure-shear specimens are widely used because the driving force can be made approximately independent of crack length over a defined central region (Figure 3a). The configuration is also the basis of the standard test method ISO 27727, which obtains the crack growth rate at several tearing energies by varying the strain amplitude [37]. With an uncracked specimen used to determine the strain-energy density, the tearing energy is written as

*T* = *Wh*, (3)

where *W* is the strain-energy density at the imposed deformation and *h* the undeformed specimen height. Some papers use the formally analogous *G* = *Uh*_0_, with *U* the strain-energy density as each study defines it. Formal similarity does not establish numerical equivalence, which also requires the loading or unloading branch, the conditioning history and the definition of *W* or *U* to agree. The configuration lets a crack advance under a nominally constant driving force and, in suitable protocols, yields several stabilised rate measurements without recalculating a crack-length-dependent geometry factor. However, its field is an approximation. Kocjan et al. showed by digital image correlation (DIC) a measurable length-direction contraction in a planar-tension coupon [38]. Stoček et al. found the contraction to be non-zero across the whole horizontal axis over aspect ratios 1/2–10 and strains to 0.5, and derived parametric functions for the near-pure-shear width and the minimum notch length required [39].

The specimens used in published pure-shear studies vary substantially in dimensions and procedure. Kaang et al. used 2 × 200 × 20 mm specimens with an approximately 30 mm edge cut and calculated *G* = *Uh*_0_ from the uncut force–displacement response [32], and Soma et al. used the same section dimensions for thermally aged carbon-black-filled NR [33]. Muñoz et al. used a 20 mm high, 220 mm long specimen with a 30 mm precut, cycle-resolved imaging and post-mortem fractography [40]. Narynbek Ulu et al. used a mini-pure-shear geometry with two in situ 3 mm edge notches to test a matrix of hydrogenated acrylonitrile–butadiene rubber (HNBR) formulations at 120 °C [41]. All are recognisably pure-shear experiments, but they are not interchangeable replicates.

Crack-length independence holds only within bounds, which Rivlin and Thomas set by requiring both the specimen width and the cut length to be large compared with the unstrained height between the grips [13]. The lower bound follows from the derivation, which needs a fully relaxed region behind the crack and a pure-shear region ahead of it. If the cut is short relative to the height, the transition zone occupies the whole region behind the crack. In the pure-shear specimens above for which dimensions are reported, the ratio of cut length to unstrained height lies between about 1.5 and 2.5. Because Rivlin and Thomas stated the bound qualitatively, these ratios cannot be assumed to satisfy it without a specimen-specific check such as that of Stoček et al. [39].

The upper bound requires the crack tip to remain within the region approximating the intended field. Wang et al. used a 140 × 10 × 2 mm specimen with a 25 mm initial crack and avoided regions close to the free boundaries [34], and Muñoz et al. treated the late test stage separately once the remaining ligament no longer supported the approximation [40]. A report of *T* = *Wh* without specimen dimensions, admissible crack-length window and crack path is insufficient for quantitative reuse.

A study may also use the pure-shear configuration without *T* = *Wh*. Tunnicliffe analysed a carbon-black-filled NR experiment in plane-strain pure shear with an expression that compensates for the loss of load-bearing cross-section as the crack extends and for the strain energy remaining in the cracked part, which makes the driver depend explicitly on crack length [36]. The resulting power-law coefficients serve as a controlled within-study comparison across carbon-black grades and are kept separate from coefficients obtained with an uncorrected *T* = *Wh* evaluation. Pure shear thus simplifies one part of the measurement layer (the crack-length dependence of the nominal driving force), and only where geometry and boundary conditions remain valid.

### 4.2. Single-Edge-Notched Tension Specimens

Single-edge-notched tension (SENT) specimens are attractive for simple loading, parallel testing of several specimens and a direct link to tensile fatigue. Their energy release depends on the evolving crack length and a geometry correction. El Maanaoui and Meier tested simultaneous SENT specimens and expressed the driving force as *T* = 2*πkW*_tot_*c*, with *c* the crack length and $k=1/\sqrt{1+\varepsilon }$ a strain-dependent geometry factor [42]. Such a factor depends on the extension as well as on the configuration. In Greensmith’s original measurements, the corresponding quantity fell from about 3 at small extensions to somewhat below 2 at 200% extension [14]. An analysis that instead uses a fixed value of *k* for tearing energies obtained at different strain amplitudes distorts the horizontal axis systematically. Because the strain needed to reach a given tearing energy changes with temperature, the same distortion can enter temperature comparisons unless *k* is re-evaluated at each condition. Monotonic single-edge-notched (SEN) studies add that the inferred onset energy depends on finite-viscoelastic size effects and on the view used to locate initiation [43,44].

SENT data are most valuable when crack length, geometry factor and minimum load are measured with adequate resolution, and they bridge towards finite geometries in which the driving force changes as the crack grows. However, their coefficients remain SENT results, and combining SENT and pure-shear data requires first showing that the energy definitions and constraint states are compatible over the compared range.

### 4.3. Double-Cantilever-Beam, Trouser and Other Geometries

Alternative geometries access a different constraint state, crack velocity or experimental range, and so interrogate a different part of the fracture response. In an instructive case, South et al. obtained a nominal plane-strain response from a modified double-cantilever-beam (DCB) configuration and a nominal plane-stress response from an SEN specimen, and on their energy scale, the DCB rate was approximately two orders of magnitude faster [26]. The two tests also differed in frequency, at 2 and 0.25 Hz, and used two driving-force calculations, Δ*G* and a Rivlin–Thomas construction. Constraint acts through two paths. In the linear-elastic relation conventionally used for the conversion, energy release rates under plane stress and plane strain differ by a factor 1 − *ν*^2^, approaching 0.75 in the incompressible limit, which is far too small to produce two orders of magnitude. The second path is the crack tip stress state, where high triaxial tension can admit cavitation and alter the conditions for strain-induced crystallisation. Only this second path remains a plausible large contributor. However, neither path was measured. The large contrast therefore reflects the bundled differences between the two tests and provides no fixed plane-strain factor.

Trouser tearing provides another window, particularly at high crack velocity or for steady tearing, with its own force partition, leg deformation and dissipation fields. In the Rivlin–Thomas analysis, the trouser tearing energy is *T* = 2*λF*/*t* − 2*wW*, with *F* the applied force, *λ* the extension ratio of the legs, *w* the leg width and *W* the strain-energy density stored in them, reducing to *T* = 2*F*/*t* only when the legs are effectively inextensible [13]. A trouser value may not even be single-valued. De and Gent found a filled SBR at 70 °C tearing knotty under an intermittent force. Its trouser tearing energy, evaluated on the uncorrected *T* = 2*F*/*t* basis, therefore had to be given as a maximum, average and minimum of 44.0, 28.4 and 12.7 kJ m^−2^ [45]. The choice among these numbers in a later comparison is also part of the energy convention. Duncan et al. combined pure-shear crack growth and trouser-tear datasets in one continuous model for non-strain-crystallising rubbers [21]. Each dataset, however, retains its geometry and thermal history.

A different geometry thus changes the relation between remote deformation, crack tip fields, dissipative volume and reported energy release, and it forms part of the definition of the independent variable instead of serving as an alternative fixture for the same scalar property.

### 4.4. Energy Accounting

Even with geometry fixed, the value on the horizontal axis depends on how strain energy is calculated. Because rubber shows hysteresis, stress softening, permanent set and temperature-dependent viscoelasticity, the area under a loading curve, the recoverable area on unloading, the full hysteresis-loop area and an equilibrium stored-energy estimate answer different questions (Figure 3b). The branches are not symmetric alternatives. The tearing energy is defined on stored elastic energy, whereas the integral of the loading curve contains both stored and dissipated contributions. The loading-branch value is therefore the larger, and the difference is the bulk hysteresis. According to Tunnicliffe, the unloading branch has been the more common choice in published FCG work [36].

The pure-shear studies characterised the strain-energy density in different ways. Kaang et al. calculated it from the load–displacement response of an uncut specimen at each tested strain [32], and Narynbek Ulu et al. integrated the nominal stress–stretch response of an uncracked mini-pure-shear specimen after conditioning [41]. Rublon and Favier preconditioned for 300 cycles at 50% global strain and 5 Hz and then characterised uncracked specimens over 5–50% strain before notching [46]. These procedures reduce some transient effects but do not make their *W* values equivalent.

Tunnicliffe quantified the consequence by calculating tearing energy from both loading (*T*_L_) and unloading (*T*_UL_) branches and fitting power-law parameters separately [36]. Across the compound set, the fitted exponent was substantially unaffected by the branch, whereas the intercept moved systematically. The coefficients therefore remain branch-paired, and combining an intercept from one branch with an exponent from the other would produce a relation that was never measured. Yamabe et al., testing eleven SBR vulcanisates in pure shear at 5 Hz and a strain ratio of 0.1, computed the energy-density range from the loading portion of the stabilised cyclic curve, gave the unloading range alongside it, and defined a hysteresis ratio HR = 1−Δ*T*_UL_/Δ*T*_L_ [27]. Both branches gave the same power-law exponent in the fitted tearing-energy–strain relation, so the hysteresis ratio was independent of strain amplitude over the investigated range. A branch difference is thus a systematic horizontal offset, of the size of the compound’s hysteresis, and differs in kind from random scatter. A recoverable-energy route that separated unloading work from total input energy combined monotonic cracked tests with lower-rate uncracked and simple-shear data, and its correction is therefore an accounting construction obtained without a cyclic crack-growth measurement [47].

The hysteresis-loop area may correlate with heat generation, damage or fatigue life, but it is not the crack-driving energy of a Rivlin–Thomas expression. Studies of unnotched fatigue that use dissipated energy as a predictor address an endpoint distinct from pre-cracked d*c*/d*N*, and the two stay numerically separate unless a derivation and a validation bridge them. A horizontal shift in a d*c*/d*N*–*T* curve can be assigned to formulation only when the report states the characterisation specimen and cycle; the branch; the treatment of residual strain; and whether the value is total work, recoverable energy or dissipation.

### 4.5. Crack Preparation and Conditioning

A razor cut can differ in tip radius, residual stress and local damage from a naturally propagated fatigue crack, and the early measured rate may therefore describe notch conditioning rather than the steady material response. Using an ethylene–propylene–diene rubber (EPDM) reference, Ryzí et al. found that blade-tip radius, repeated use of an uncoated blade and specimen orientation dominated the measured intrinsic cutting energy, whereas relaxation time and the number of strain levels were negligible only under that material-specific protocol [48].

Several protocols therefore include a conditioning stage before rate data are accepted. Rublon and Favier inserted two 5 mm edge notches and applied a short preliminary cyclic sequence to turn the incisions into fatigue cracks [46]. Narynbek Ulu et al. conditioned mini-pure-shear specimens at 2.5 Hz until the cycle-to-cycle change in tearing energy fell below 1%, performed the energetic characterisation, introduced the notches in situ and then recorded stabilised rates [41]. Wang et al. excluded the first 1 mm of extension, over which a rapid-to-slow transient followed the initially sharp cut, before fitting crack extension against cycle number [34].

Conditioning also alters the bulk specimen through the Mullins effect, viscoelastic relaxation, permanent deformation and heat generation. It is therefore part of the material state as well as of the procedure, and the maximum prior strain, number of cycles, frequency, temperature and stabilisation criterion together determine what the subsequent FCG curve represents.

### 4.6. Imaging and Rate Extraction

Laboratories derive the crack growth rate from crack-length records in different ways. Kaang et al. tracked the crack tip in situ with a high-speed charge-coupled-device (CCD) camera and took the rate from the slope of crack length against cycle count [32]. Muñoz et al. phase-locked image acquisition to the fatigue cycles and obtained instantaneous crack velocity in place of a cycle-based Paris coefficient [40]. Rublon and Favier periodically stopped the test and measured the mean advance of two cracks with a binocular microscope [46]. Stadlbauer et al. also showed that shadow imaging could miss ligaments or out-of-plane damage that front and back illumination revealed [49]. These methods differ in temporal and spatial resolution, interruption history and statistical unit. Post-test fractography locates initiation sites and crack paths, but the fracture surface alone does not establish the chronology of growth [2]. In post-mortem scanning electron microscopy, Muñoz et al. found a rough, disordered fracture surface at very low crack velocity and sawtooth striations at high velocity, whose width tracked the in situ velocity only in striated zones [40].

For thresholds, Steck et al. observed that the steady-state rate is total crack advance divided by total cycle count, so its resolution is the crack-length resolution divided by the cycle count. Too coarse a resolution systematically overestimates the threshold, and reported resolutions vary over about three orders of magnitude [11]. Robertson et al. provided a within-programme counterpart, showing on one carbon-black-filled EPDM compound that the Lake–Yeoh cutting method returned in two hours a threshold nearly identical to one requiring 7.5 months of continuous testing [50]. A stated crack resolution and the cycle count over which non-detection was established are therefore conditions for placing a threshold on a common axis.

For coefficients, Liu et al. compared, on one compound at a common frequency and load ratio, crack length obtained by intermittent imaging of a pure-shear specimen with crack length obtained continuously from force and torque signals on a ring specimen [51]. The fits gave coefficients of 4.8933 × 10^−6^ and 4.9631 × 10^−6^ and exponents of 2.4509 and 2.4012 (relative differences of 1.42% and 2.03%), with the continuously derived points inside factor-of-two scatter bands about the imaging-based fit. Because geometry, amplitude sequencing and observation route changed together, the comparison calibrates a new configuration against an established one and does not isolate an observation effect. The same authors noted two difficulties of imaging in rubber, namely that at large deformation, the withdrawn camera loses spatial resolution and that blunting turns the tip from a point into an arc that offers no stable reference for crack length.

**Figure 4:**
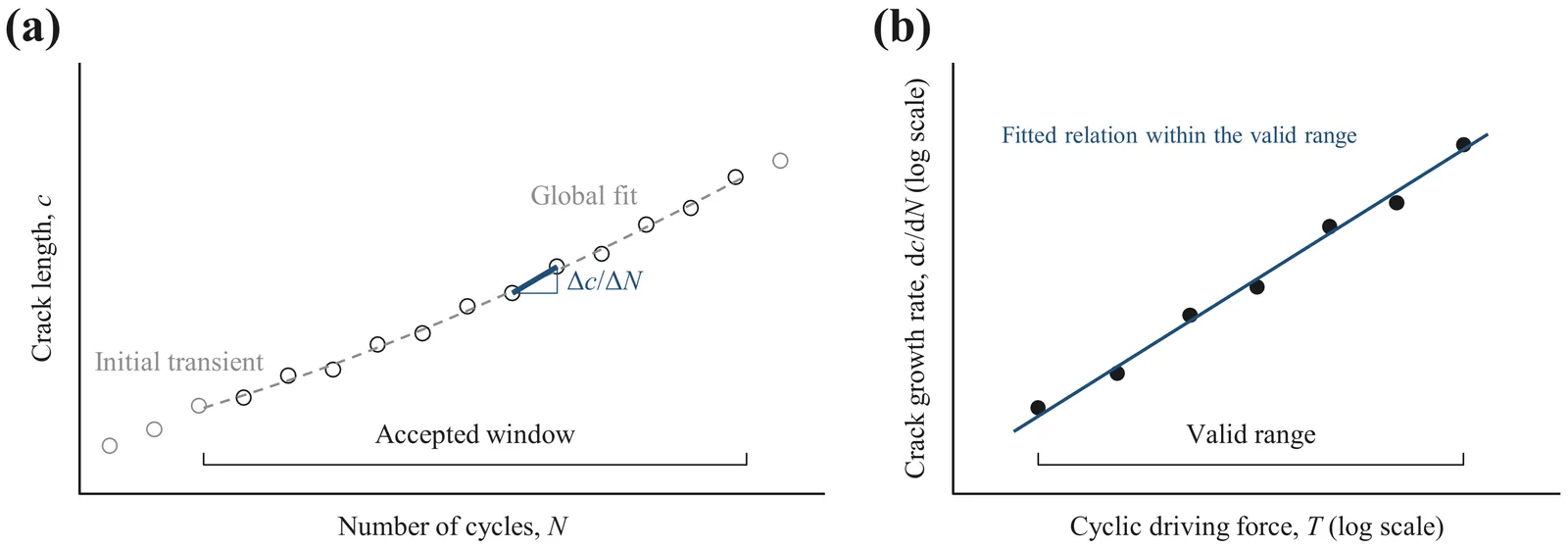
Reduction of a crack-length record to a crack growth rate; schematic drawn by the authors. (**a**) Crack length *c* against number of cycles *N*. Grey points in the initial transient and beyond the accepted window are excluded; a local secant gives Δ*c*/Δ*N* between neighbouring records, and a global fit (dashed) spans the accepted window. (**b**) The resulting rates d*c*/d*N* against cyclic driving force *T* on logarithmic axes, with the relation fitted within its valid range.

These methods also fix what counts as a replicate, since multiple crack fronts, repeated images or rate segments from one specimen are correlated subsamples within a single independent replicate. Under variable-amplitude loading, rate extraction also changes with cycle number. In one documented protocol, local secant and seven-point polynomial differentiation produced dispersed and sometimes negative low-energy rates, and the authors therefore fitted crack extension globally and differentiated the fit (Figure 4) [34,35]. Processed rates remain interpretable only while they can be traced to raw crack coordinates and cycle counts, with the fitting function, window, excluded transients, calibration, stopping intervals and specimen hierarchy preserved.

### 4.7. Shared Datasets

Several publications share an experimental ancestry and count once, since they do not provide independent confirmation of one another. The Shangguan and Wang publications describe a fully documented variable-amplitude protocol. Because they share specimens, crack histories, coefficients and validation data, they are treated as one dataset family throughout [34,35]. Similarly, the planar-tension crack growth data and coefficients for one SBR compound reappear in a later transfer study by the same group [38,52], and the Stadlbauer setup and parameter papers describe one campaign [28,49]. Two acrylonitrile–butadiene rubber (NBR) studies from one laboratory also share material, frequency and load ratio [25,53]. Much of the NBR evidence used in Section 5.4, Section 7.4 and Section 8.2 comes from that laboratory [25,53,54], narrowing the independence of the material axis even where the compounds differ.

## 5. Loading History, Thermal State and Data Reduction

**Figure 5:**
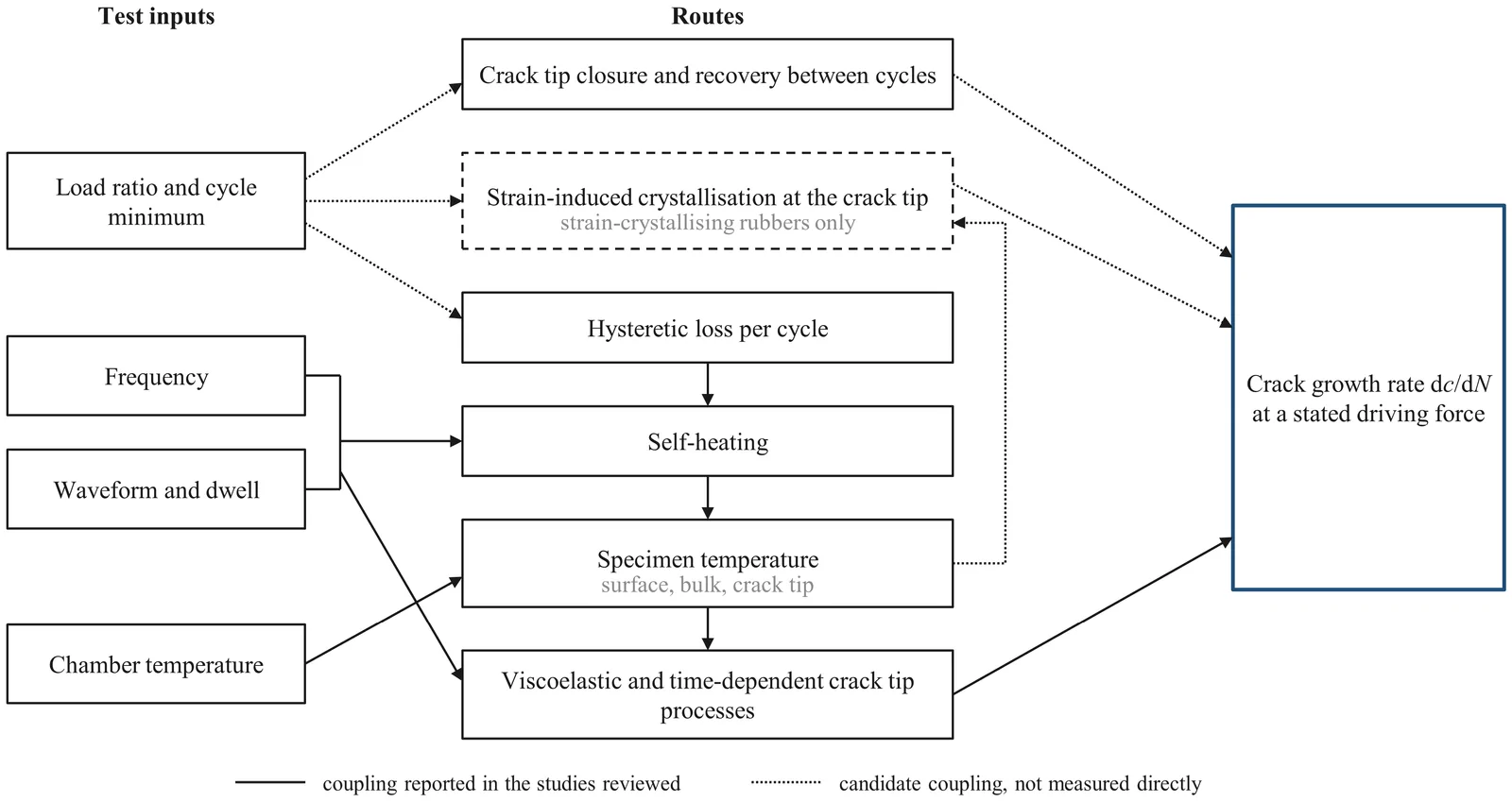
Couplings through which the loading and thermal test variables act on the measured crack growth rate; schematic drawn by the authors. The test inputs on the left, which fall under items 4–6 of the comparison checklist (Table 3), act on the crack growth rate at a stated driving force through the central routes. Solid arrows mark couplings reported in the studies reviewed and dotted arrows candidate couplings that those studies did not measure directly; the dashed box marks the route that operates only in strain-crystallising rubbers. None of the thirteen FCG studies in Table S1 recorded a specimen temperature alongside the rate, and the measurements outside that set are of a control specimen or the specimen surface (Section 5.3), so solid links through specimen temperature rest on chamber-temperature contrasts and surface measurements; prior ageing, also under item 6, acts on the material state and is not drawn. The effect of the load ratio on the rate is itself measured (Section 5.1); only the routes through which it acts are candidates. The couplings are discussed in Section 5.1, Section 5.2, Section 5.3, Section 7.1, Section 8.1, Section 8.2 and Section 8.4.

Frequency, waveform, load minimum and thermal state act on the crack tip through shared routes, chiefly self-heating; viscoelastic dissipation; and, in crystallising rubbers, strain-induced crystallisation. A change in one variable can therefore appear as the effect of another (Figure 5).

### 5.1. Load Ratio and the Cycle Minimum

**Figure 6:**
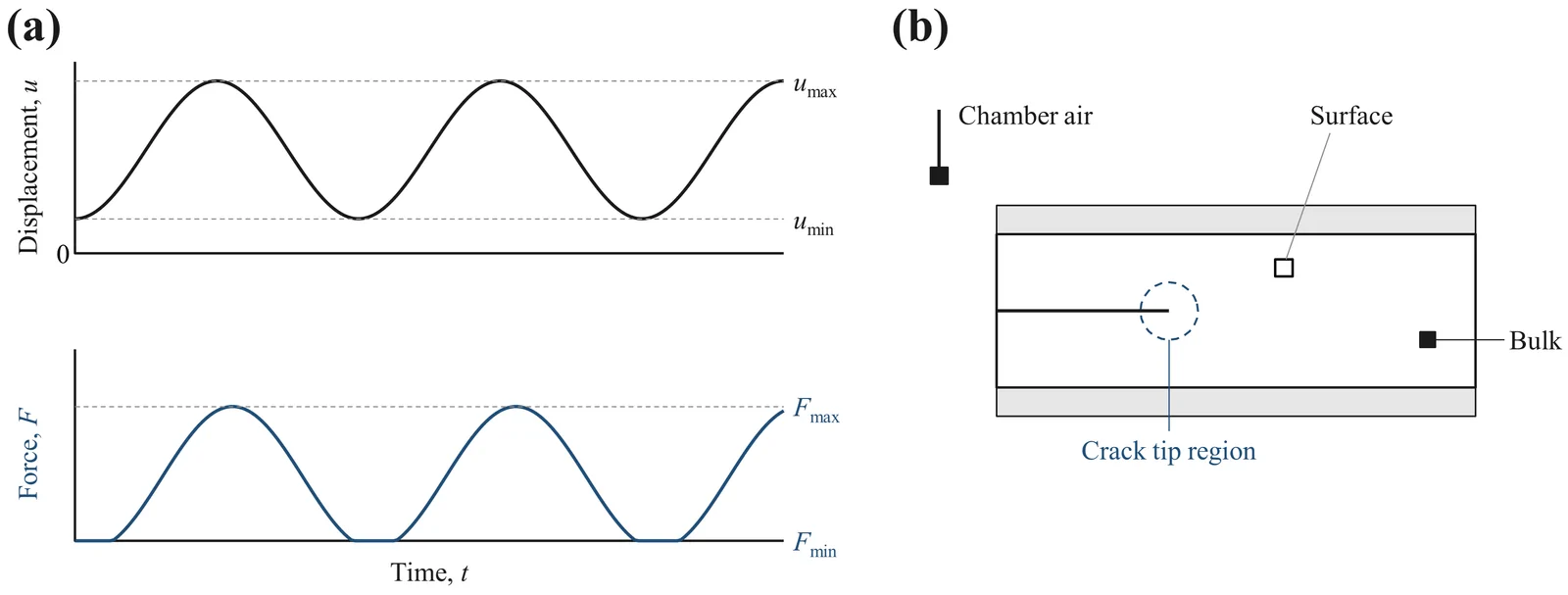
Load ratio and temperature measurement locations; schematic drawn by the authors. (**a**) A displacement-controlled history *u*(*t*), with *u* measured from the unstrained clamp separation, whose minimum *u*_min_ lies above zero, and the resulting force *F*(*t*), whose minimum *F*_min_ falls to zero over part of each cycle, so that the load ratio on displacement differs from that on force. (**b**) A pure-shear specimen containing a single crack, with the locations at which temperature can be measured: chamber air, the specimen surface (open square), the bulk (filled square) and the crack tip region (dashed circle).

The cycle minimum is left under-specified in several of the studies compared here, and its effect on the crack tip is direct. A single symbol, *R*, covers a force ratio, a displacement ratio, a strain ratio or a tearing-energy ratio *R* = *T*_min_/*T*_max_ (Figure 6a). Because tearing energy scales non-linearly with deformation, these do not map onto one another without a material-, geometry- and history-specific relation, and nominal *R* = 0 conditions can therefore represent different crack tip states. Warneboldt et al. further showed that actuator displacement or rotation ratios need not equal the local strain ratio after viscoelastic accommodation, although their macrocrack-based life endpoint provides context on the loading state and yields no FCG data [55].

The published studies use physically different minima. Kaang et al. described a fully relaxing condition with the minimum strain returned to zero [32], and Narynbek Ulu et al. used a displacement-based *R* = 0 [41]. Shangguan et al. and Wang et al. defined the ratio energetically as *R* = *T*_min_/*T*_max_ = 0 [34,35]. Hassim et al. used a minimum-load target of 1 N, a small positive force [56], and Muñoz et al. returned the force to zero at each cycle minimum under trimodal displacement control [40]. Four of the thirteen studies in Table S1 leave the ratio unresolved (Rublon and Favier, Béranger et al., El Maanaoui and Meier, and the Duncan multi-dataset model). Where a ratio is stated, its basis still differs: South et al. gave *R* = 0 in load control, Narynbek Ulu et al. and Tunnicliffe defined the ratio on displacement or strain, and Shangguan et al. on tearing energy.

A small positive minimum can change crack closure, the recovery of viscoelastic deformation, the strain-induced crystallisation retained at the crack tip and the energy dissipated per cycle. A zero displacement minimum does not guarantee a zero force minimum once the material has taken a permanent set. Treating “nearly relaxing”, “minimum force” and *R* = 0 as one category therefore pools different crack tip histories.

Legorju-Jago and Bathias showed that the minimum load is also mechanistically active in NR. Defining *R* = *F*_min_/*F*_max_, they found propagation suppressed at *R* = 0.5 for the maximum loads tested, whereas the threshold was nearly absent under alternating tension–compression at *R* = −1 [57]. Lake had earlier proposed a mechanism: if the minimum stress is sufficiently high, crystalline structure at the crack tip is at least partially retained between cycles, which can raise the threshold tearing energy *T*_0_ for crystallising rubbers, as shown for a natural rubber cycled at a tearing-energy ratio of 0.056. The weaker effect in amorphous rubbers was attributed to time-dependent mechanical crack growth [58]. Consistent with this, load ratio had no marked effect on crack growth in a filled, non-crystallising NBR, whereas lower frequency increased the rate; waveform also had no marked effect [25]. Tanaka et al. observed a comparable suppression above a displacement ratio of 0.23, although on a cyclic *J*-integral axis [29]. Li et al., in contrast, fitted an *R*-dependent Mars–Fatemi exponent over 0 < *R* < 0.3 from S–N fatigue-life data [59]. This fit therefore cannot be pooled with the direct FCG contrast of Legorju-Jago and Bathias as confirmation. Even at identical cyclic extrema, *R_ε_* = 1/3 and frequency, changing whether the preload ended at maximum or minimum displacement changed filled-NR behaviour from bounded backward growth to catastrophic propagation [60]. Separating these effects requires a matched factorial map across *R*, temperature, frequency and measured specimen temperature for strain-crystallising and non-crystallising formulations, which no reviewed study provides.

### 5.2. Frequency, Waveform and Dwell

Frequency bundles the strain rate imposed on the viscoelastic network, the dwell time available for recovery or time-dependent crack tip processes, and the rate of hysteretic heat generation. Busfield et al. separated these by splitting growth per cycle into a time-dependent component, obtained from constant-*T* static tests, and a cyclic remainder. Over 0.01–5 Hz in unfilled SBR, low frequency or high tearing energy left the time-dependent term dominant and high frequency with low energy the cyclic one, while in highly swollen SBR, the time-dependent term dominated throughout [30]. In matched pure-shear tests, Stadlbauer et al. linked apparent frequency effects to heat build-up, found slower growth at positive and faster growth at negative load ratios, and showed dwell-containing waveforms above a sinusoidal reference within the tested formulations and ranges [28].

Kaang et al. found no appreciable difference in crack growth rate between 1 and 10 Hz for their carbon-black-filled NR and SBR at 40 and 100 °C [32]. Narynbek Ulu et al. likewise resolved no frequency effect at 2.5, 10 and 24.5 Hz for HNBR at 120 °C [41]. Such null results need care because neither study carries the specimen-temperature record or statistical power needed to establish a null. Frequency can also serve as a thermal control. Champy et al. used 0.75–8 Hz to keep heat build-up in the same order of magnitude across NR/polyisoprene (IR) loading conditions, although their endpoint was stiffness-loss life [61].

Frequency is further entangled with the conditioning that precedes rate measurement. Tunnicliffe preconditioned for 10,000 cycles, Rublon and Favier for 300 cycles at 50% strain and 5 Hz, and Narynbek Ulu et al. at 2.5 Hz to a 1% stabilisation criterion before switching to 24.5 Hz (Table S1). The network state entering the measurement therefore differs across protocols.

The energy dependence of the rate also departs from a single power law. Duncan et al. represented the response of non-strain-crystallising rubbers as a sum of slow and fast contributions, *T* = *T*_s_ + *T*_f_, in which the slow term is capped at a threshold tearing energy *T*_d_, so that no separate transition point for fast growth is needed [21]. A frequency change that is immaterial in one energy range therefore need not be immaterial in another.

Waveform and dwell are often undocumented in the studies compared here. Table S1 names a sinusoid for four of its thirteen studies. El Maanaoui and Meier specified 50 ms pulses at 10 Hz without naming the pulse function [42], and no waveform is given for the remaining eight. The omission matters. Liu et al. found that at 20 Hz in silica-filled solution SBR (S-SBR), two pulse histories produced greater surface heat build-up and faster pre-cracked growth than a sine history. The shorter excitation fraction produced the largest effect under the tested displacement-controlled *R* = 0 condition [31]. Extrapolating a laboratory frequency to service therefore requires the actual specimen temperature because self-heating is expected to grow with frequency and can turn an apparent frequency effect into a temperature effect.

### 5.3. Test Temperature Versus Specimen Temperature

In all thirteen studies in Table S1, the temperature given for the crack growth measurement is a chamber or ambient condition, and no specimen temperature measured during the test accompanies it. Outside that set, in contrast, Kyei-Manu et al. measured the temperature of a control specimen with a contact thermocouple and found a rise of 0.5–0.6 °C at the displacement where most of a step change in growth rate occurred [62]. The chamber set point, the specimen surface temperature, the bulk temperature and the steady-state temperature reached under cyclic self-heating are four distinct quantities. These can differ substantially, and the difference would be expected to widen with frequency, strain amplitude, filler loading and specimen thickness.

Kaang et al. tested at 40 and 100 °C; El Maanaoui and Meier at chamber temperatures of 23, 60 and 90 °C; and Rublon and Favier at room temperature, 80 °C and 100 °C, in each case as the controlled environment [32,42,46]. Narynbek Ulu et al. held the specimen surface at 120 °C under a pyrometer only in their fatigue-life experiments, whereas their crack-propagation tests give the chamber set point [41]. A crack growth law calibrated against a chamber set point but governed by a higher, unrecorded crack tip temperature misattributes a thermal effect to the nominal test temperature. Any transfer of that law to a component with a different thermal balance inherits the error.

The gap also varies within a study because it is driven by the same loading and material variables the experiment is trying to isolate. In the South et al. comparison discussed above, the DCB test at 2 Hz and the SEN test at 0.25 Hz differ by nearly an order of magnitude in frequency. Frequency-dependent self-heating may therefore account for part of the rate contrast that South et al. attributed to plane-strain constraint. Where self-heating is possible, the thermal state of the data is defined by the steady-state specimen temperature instead of the chamber temperature (Figure 6b).

### 5.4. Constant Versus Variable Amplitude

Under constant-amplitude loading of a pure-shear specimen, the driving force stays approximately constant over the admissible crack-length window, crack length increases approximately linearly with cycle number and one stabilised rate follows at each tearing-energy level. Variable-amplitude loading breaks that simplicity because the maximum tearing energy evolves from cycle to cycle. For 42 phr carbon-black-filled NBR at 4 Hz and *R_ε_* = 0.5, Schieppati et al. found that increasing histories gave essentially the same stable curve, whereas decreasing histories gave slightly faster growth, especially at low energies outside the stable Paris-fit regime [25].

The Shangguan/Wang dataset family shows how variable-amplitude data can be handled [34,35]. In the edge-notched pure-shear coupon of Section 4.1, the strain-energy density came from the unloading curve and the peak strain increased monotonically from 0 to 0.75. After excluding the initial transient and differentiating a global power-function fit of crack extension against cycles, the authors paired the rate with the evolving maximum tearing energy, restricted the fit to maximum tearing energies below 2100 J m^−2^ and obtained

(4) $$\frac{dc}{dN}\;[{\mathrm{mm cycle}}^{-1}]=4.65802\times 10^{-12}\;(T_{\max}\;[{\mathrm{J m}}^{-2}]{)}^{2.33362},\;\;r^{2}=0.9678.$$

The relation applies to this material, these units, a sinusoidal *R* = 0 cycle with monotonically increasing peak strain, and *T*_max_ < 2100 J m^−2^. The rate points derive from three physical specimens (s4–s6) and are not independent replicates. The 2100 J m^−2^ bound is a model-exclusion limit chosen by the authors, not a measured thermal threshold.

Even a constant-amplitude test does not always yield a single-valued rate. Muñoz et al., measuring instantaneous crack velocity at 1 Hz and room temperature, observed the high-energy response alternating between two velocity states linked to a sawtooth striation morphology [40]. Because a rate is constructed over an averaging window, an unstable or multi-valued response should be reported as such.

### 5.5. Data Reduction, Uncertainty and Reproducibility

The global fit of the Shangguan/Wang dataset family avoided negative rates but imposed a functional form and correlated the derived points [34,35]. The window, excluded transients and fitted function therefore have to be reported so that the curve can be reconstructed. How much depends on that record is visible when a group compares its data with the classical literature. Yamabe et al. placed their unfilled SBR results beside the earlier unfilled-SBR datasets of Lake and Lindley [16] and Busfield et al. [30], recording those test frequencies as 1.7–2.2 Hz and 5 Hz. They found that the transition region described in the earlier of the two did not appear in their data and that their threshold was somewhat higher [27]. The authors attributed the discrepancy most probably to the data-acquisition procedure of their strain-decreasing mode, with formulation differences secondary.

Uncertainty also has to be traced to the correct statistical unit. A defensible hierarchy distinguishes, in decreasing order of independence, separate specimens, multiple crack fronts or repeated images within one specimen, and multiple points read from a single fitted curve. Rublon and Favier measured two crack tips on one specimen per formulation [46], and the Shangguan/Wang points share one fitted curve [34,35]. Most of the remaining studies do not state the hierarchy.

In crack-length-dependent geometries, the crack length enters both axes of the rate–energy plot. For SENT and similar configurations, the driving force is computed from the crack length through a geometry factor, and a measurement error therefore moves both the crack-extension increment and the calculated tearing energy. Ordinary regression, which assigns all scatter to the ordinate, is ill posed for such data, and imaging resolution and calibration are part of the uncertainty budget. Pure-shear configurations are less exposed to this coupling inside their crack-length-independent window.

## 6. Reporting and Applying the Checklist

### 6.1. A Minimum Reporting Set

The checklist of Section 3.5 can only be applied to the information that primary reports supply. Table 4 proposes a minimum reporting set in eight domains, from material state to endpoint, transfer and validation, derived from the ambiguities that recur in Section 4 and Section 5. It is intended for use alongside test standards such as ISO 27727 [37] and lists what a report needs to contain for its results to be compared with those of another laboratory.

For a laboratory planning a formulation ranking that others can reuse, several practices go beyond recording the items of Table 4. The tearing energy should be reported from both the loading and the unloading branch, or the branch used should be stated, together with how *W* was characterised. The measured minimum force should be recorded alongside the programmed minimum, and the specimen temperature measured, at least at the surface, instead of reporting only the chamber set point. The raw crack-length–cycle data should be archived with the fitting window and function, so that rates can be recomputed, and the near-pure-shear window should be checked for the specimen proportions used [39].

### 6.2. Applying the Checklist

Table 5 collects six cases from the reviewed literature in which naming the unresolved item changes the supported conclusion. In each, an apparently general statement resolves into a narrower, condition-specific one.

**Figure 7:**
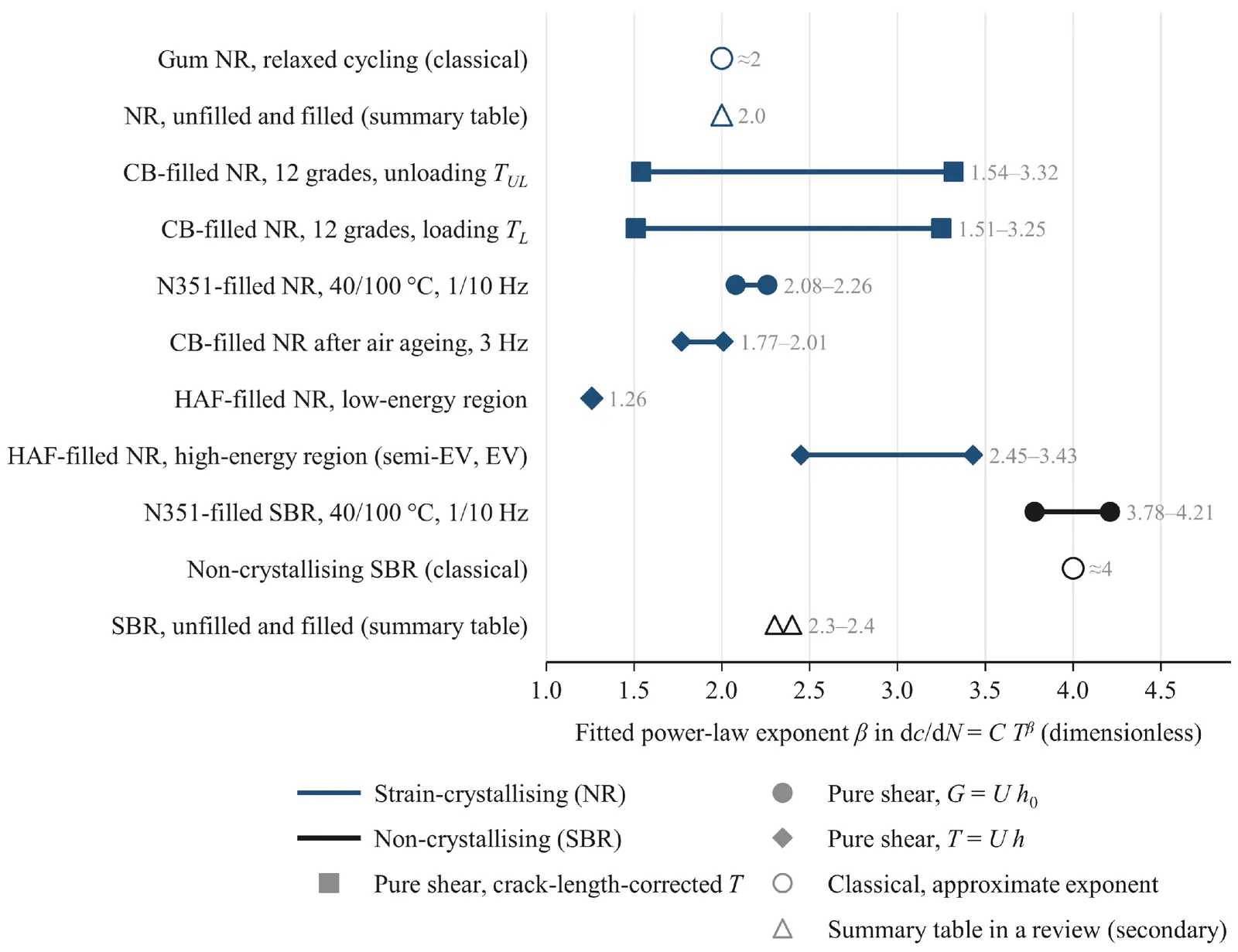
Fitted power-law exponents *β* in d*c*/d*N* = *C* *T^β^* reported for NR and SBR in different programmes. Each bar or marker is the range or value reported within one source, and values are not pooled; marker shape identifies the driving-force construction or, for open markers, the type of source, and colour separates strain-crystallising NR from non-crystallising SBR. Carbon-black-filled NR with twelve grades at constant hardness, unloading- and loading-branch tearing energy [36]; N351-filled NR and SBR in one pure-shear programme at 40 and 100 °C and 1 and 10 Hz, tabulated values [32]; carbon-black-filled NR after air ageing [33]; HAF-filled NR before ageing, the low-energy region of a two-region fit shared by three cure systems and its high-energy region for the semi-efficient and efficient cure systems [69]; approximate classical exponents for gum NR and non-crystallising SBR [17,18,19]; and a summary table in an earlier review, attributed there to Gent [3]. In the legend, *U* is the strain-energy density, written *W* in Equation (3). Plotted by the authors from the values given in the sources’ text and tables. Values of Andreini et al. [68], given in the text, are not plotted. The figure shows the spread of reported values; it is not a comparison admitted by the checklist of Table 3.

The classical contrast between strain-crystallising and non-crystallising rubbers shows how the checklist works on a positive claim (Figure 7). Within one programme, Kaang et al. tested N351-filled NR and SBR in the same pure-shear geometry, with the same filler, loading and energy definition, and obtained tabulated exponents of 2.08–2.26 for NR and 3.78–4.21 for SBR [32]. The tabulated NR exponents are 2.08, 2.26, 2.13 and 2.17 for the four temperature–frequency cells, whereas the text of the paper gives 2.08–2.13. The corresponding SBR values are 3.78, 4.21, 3.97 and 3.84. Tunnicliffe’s twelve carbon-black grades in one NR matrix gave 1.54–3.32 on the unloading branch [36]. Against Kaang’s programme, however, the driver construction (a crack-length-corrected plane-strain pure-shear expression versus *G* = *Uh*_0_), the frequency (2.5 Hz versus 1 and 10 Hz) and the test temperature (room temperature versus 40 and 100 °C) differ. Specimen dimensions and preconditioning (10,000 cycles against none reported) also cannot be shown to match. The replicate hierarchy cannot be matched either because Tunnicliffe tested in duplicate whereas Kaang et al. gave no specimen count. Items 2, 3 and 5–7 are therefore unresolved, and the two sets of exponents cannot be compared quantitatively.

The comparison therefore cannot show whether the ordering between the two rubbers is general. The sources that give exponents for both rubbers place NR below SBR in all but one case. They are Kaang’s programme; Andreini et al., whose pure-shear exponents were 2.03 and 2.09 for NR against 2.50 and 2.85 for SBR, although single-edge-notched specimens, which the authors judged less reliable, gave 3.01 for NR against 2.28 for SBR under sinusoidal loading [68]; and a summary table in an earlier review, attributed there to Gent, that lists exponents of 2.3 and 2.4 for unfilled and filled SBR against 2.0 for NR [3]. The values, however, do not transfer between programmes. In NR filled with high-abrasion-furnace (HAF) black and cured with an efficient-vulcanisation system, the high-energy slope reached 3.43 before ageing [69], above the upper end of Tunnicliffe’s twelve-grade range and above the 2.0 that the summary table lists for NR. The classical *T*^2^ and *T*^4^ relations [17,18,19] therefore describe particular regimes and are not constants of either polymer. The lower exponent of NR relative to SBR is an ordering seen in most of these values, none from a study that isolated the backbone as its sole cause. Exponents support a numerical comparison only when measured under matched test conditions, or within bounds measured by a within-study contrast on the same base polymer, even where each source shows the same ordering (Figure 7).

## 7. Material Architecture and Crack Tip Mechanisms

With the measurement layer fixed, residual differences in FCG can be read against material architecture. For each formulation lever discussed below, Table 6 summarises the within-study effect, the conditions, where the effect reverses or remains unresolved, and the evidence level (controlled, C; partially controlled, P; exploratory, E).

### 7.1. Polymer Backbone and Strain-Induced Crystallisation

The sharpest architectural distinction is between a strain-crystallising rubber such as NR and non-crystallising rubbers such as SBR, NBR or EPDM. The distinction is real, but polymer type is entangled with filler, crosslinking and temperature in the studies reviewed here, and a bare “NR beats SBR” statement is therefore not a defensible material ranking.

Kaang et al.’s N351-filled NR and SBR programme, whose exponents were compared in Section 6.2, tested both rubbers in the same pure-shear geometry [32]. Because geometry, filler grade and loading were matched, this is a paired within-study contrast, although it does not isolate the backbone as the sole cause. Andreini et al. also compared SBR, NR and BR at a fixed 50 phr N330. However, their central variables were the specimen and the waveform, and minor adjustments for comparable curing rates mean that the backbone was not isolated [68].

A leading mechanism for the condition-dependent resistance of NR is strain-induced crystallisation (SIC) at the crack tip, commonly described through crystallisation and melting kinetics [73]. The Kaang contrast did not measure SIC. Separate studies have shown that crystallinity at a moving crack tip can be lower than the static value at the same stretch [74] and have mapped a multiaxial crystalline zone at a reduced 0.1 Hz acquisition frequency after nominal 2 Hz cycling [75]. Others have observed crack arrest or deviation under severe relaxing loading [76], imaged crack branching in situ as an energy-dissipating resistance mechanism [77] and related post-mortem striation morphology to SIC [78]. Outside NR, Narynbek Ulu et al. observed multi-cracking and out-of-plane path changes in fatigue-life specimens of their HNBR blends [41]. These are separate studies that do not form one causal sequence, and filler debonding, cavitation and hysteretic heating remain possible contributors unless measured in the same dataset.

The mechanism evidence also remains endpoint-bound. Paired micro-DIC and wide-angle X-ray diffraction (WAXD) on a stationary pre-notched unfilled-NR specimen linked local crystallinity mainly to the major principal true strain over the sampled biaxiality range, while an unprecracked diabolo life study linked branching and hardening to proposed cumulative SIC. Neither measured crack tip crystallinity during FCG [79,80].

### 7.2. Carbon Black: Surface Area, Structure and Strain Amplification

Carbon black changes FCG through reinforcement, strain amplification at the crack tip and additional dissipation, but its effect is not a single monotone function of surface area. The clearest within-study anchor is Tunnicliffe’s plane-strain pure-shear study of one NR compound reinforced with twelve carbon-black grades [36]. Because the series was compounded to a common hardness, the loading was adjusted from 39 to 108 phr across the grades, and grade and filler volume fraction move together by design. With loading and unloading branches analysed separately, the exponent ranged from 1.54 to 3.32 for *T*_UL_ and from 1.51 to 3.25 for *T*_L_, while the strain-amplification factor ranged from 2.93 to 3.90. The exponent decreased as the calculated strain amplification increased, which Tunnicliffe attributed to enhanced strain amplification promoting SIC in the crack tip region, while compound hysteresis was associated with the horizontal position of the curve through the intercept. Because all twelve compounds share one matrix and one set of test conditions, and the fitted exponent was insensitive to the energy branch, they support a structure–property comparison.

Particle size and structure do not act monotonically either. In a constant-hardness, displacement-controlled fatigue-to-failure programme, Kim and Jeong found a life ranking of N330 > N990 > N650 [81]. Matching hardness across grades of different reinforcing power generally requires different filler loadings, so filler volume fraction, hysteresis and self-heating also vary across the series. The ordering therefore combines grade, loading and dissipation, and it concerns a life endpoint instead of a d*c*/d*N*–*T* result. Within one 50 phr carbon-black/NR matrix, Chanda et al. found that smaller-particle and higher-structure grades grew more slowly, but the separation narrowed above about 25% strain, especially at 70 and 100 °C, preventing a temperature-independent carbon-black ranking [70]. In another pure-shear study of carbon-black-filled NR, Kyei-Manu et al. found that high-structure grades grew up to two orders of magnitude more slowly than low-structure grades until an abrupt step change near 2500 J m^−2^, above which the compounds converged. They attributed the step to strain-induced crystallisation and left open whether its position is a material property or a result of the protocol [62].

A filler-loading series shows how far the reduction step can reach into an architectural conclusion. Yamabe et al. tested carbon-black- and silica-filled SBR over 5–25 vol% filler in pure shear and found the crack growth data departing from a single power law at high tearing energy once filler content exceeded 15 vol%. They suggested that this departure reflects a transition from cycle-dependent to time-dependent growth, identified a compound-specific upper energy Δ*T*_L,C_, and fitted the exponent over all data and over the data below that bound [27]. With all data included, the exponent passed through a local minimum near 25 phr, which Yamabe et al. noted differs from the 40 phr that Klüppel had found earlier for comparable filled SBR [82]. With the high-energy data excluded, it decreased monotonically towards an asymptote. The authors attributed the minimum to data above Δ*T*_L,C_ entering the fit, noting that Δ*T*_L,C_ fell as filler content rose, so more heavily filled compounds contributed proportionally more high-energy points. Whether the exponent depends monotonically on filler loading is thus settled by the experiment together with the chosen fitting range, which is why the data-reduction check of the comparison checklist asks for the range before a coefficient is reused.

The endpoint again bounds the evidence in the SBR programme of Roche and Perier, in which poorer carbon-black dispersion strongly changed the fatigue-to-failure result but produced little separation in the energy-controlled pre-cracked propagation comparison. The formulation ranking therefore depended on whether life or stable growth was measured [83]. Near-equilibrium threshold tear strength rose with filler loading and falling particle size, from about 80 to 1200 J m^−2^ between gum and filled NR and SBR [84], and ordinary tear strength of filled compounds runs five to ten times that of unfilled ones [45], the latter a macroscopic fracture resistance on its own scale. Robertson et al. noted that the filler dependence of a threshold runs in opposite directions on the swollen-tear and cutting routes, although the swollen-tear side is a replot of the same data and provides no independent measurement [50].

### 7.3. Silica and Hybrid Filler Networks

Silica differs from carbon black less by the presence of a coupling agent than by the filler network it forms. A polar silica surface favours filler–filler association and raises hysteresis, while a silane such as bis[3-(triethoxysilyl)propyl] tetrasulfide (TESPT, Si69) converts part of that interaction into filler–polymer bonding. Klüppel observed this in one S-SBR series, in which silane-treated silica gave smaller clusters, weaker filler–filler bonds and lower hysteresis than carbon black. He also found the fitted Paris exponent first falling and then rising with filler content, which he related to crack tip flash heating [82]. As the filler-loading series discussed above shows, a minimum of this kind can also arise from the choice of fitting range, and the two accounts have not yet been separated in the studies reviewed here.

Other silica studies inform the mechanism but yield no FCG coefficients. Muñoz et al. observed, for a 50 phr silica-filled NR at 1 Hz, a multistable crack velocity linked to a sawtooth striation morphology [40]. Dong et al. characterised silica/carbon-black/NR composites through a *J*-integral analysis, reporting *J*_IC_ and a tearing modulus, and in fatigue tests at constant tensile strain, found slower cyclic crack growth at higher silica/carbon-black ratios [85]. Neither result is a d*c*/d*N*–*T* relation because the fatigue endpoint is fixed-strain and the path independence on which the *J* formulation relies is established for elastic response, which hysteresis, stress softening and self-heating in filled rubber remove.

Yamabe et al. supplied a matched silica–carbon-black comparison, holding the base SBR, the sulphur cure and the test protocol fixed while varying filler type and volume fraction. The silica series carried a TESPT silane, which the carbon-black series did not [27]. The threshold tearing energy of both families rose with loading and levelled off above about 15 vol%, stabilising near 170 and 500 N m^−1^ (equivalently J m^−2^) for the carbon-black and silica series at about 20 vol% and above, a roughly threefold separation at comparable hysteresis ratios. Four conditions travel with that number. First, it contrasts two filler systems as compounded, and the filler surfaces are not isolated. The tearing energy is the loading-branch value at 5 Hz and a strain ratio of 0.1. The threshold is the energy at which crack extension stayed below the 20 µm resolution of the microscope after 2 × 10^6^ cycles. Finally, Yamabe et al. noted that their specimen is not strictly pure shear and may be closer to plane strain, restricting measurement to a finite-element-validated central band.

Hybrid silica/carbon-black networks remain an evidence gap for d*c*/d*N*–*T* data, since the only hybrid study in the reviewed set gives *J*-integral quantities and fixed-strain fatigue growth. In a separate, non-hybrid comparison of matched 40/60 phr silica- and carbon-black-filled NR, Schneider et al. found that crack-contour propagation increased with chamber temperature, decreased with strain-based load ratio and tended to be lower with silica. Their fixed-strain endpoint, however, restricts the result to these directions [86].

### 7.4. Crosslink Density, Network Topology and Additives

Cure system and network structure also shape the fatigue response, and in the De Mattia-type fatigue-to-failure comparison of El-Sabbagh et al., peroxide-cured NR ranked highest among the cure systems tested [87]. Saeed et al. showed that changing curative level and blooming alters both the surface and the measured cyclic crack growth [71]. Kim and Lee supply a matched-density contrast before ageing [69]. Conventional, semi-efficient (semi-EV) and efficient-vulcanisation (EV) cure systems in one NR at crosslink densities within ±5% gave indistinguishable unfilled pure-shear rates on one two-region fit. With 40 phr of HAF black, the three shared a low-strain slope of 1.26 but separated at high strain, reaching 2.45 for semi-EV and 3.43 for EV. Those tests were strain-controlled at 15 Hz over 23–85% strain, and Kim and Lee found a different ordering after ageing.

Backbone modification within one polymer family provides the cleanest network anchor. Narynbek Ulu et al. studied six HNBR blends at a fixed 70 phr N772 and peroxide cure, varying acrylonitrile (ACN) content (24/36/44 wt%) and hydrogenation (91/96/>99.5%) and testing at 120 °C in a mini-pure-shear geometry over *T* = 50–1000 J m^−2^ [41]. Within that window, the A36H96 blend (labelled by ACN content and hydrogenation level) was the best. At 50 J m^−2^, the rate was about 7 nm/cycle for A36H91 against about 0.2 nm/cycle for A36H96 and A36H99, a factor of about 35. The matrix separates ACN and hydrogenation within the tested compositions. A paired NBR programme linked crack growth resistance across mould-temperature and cure-history conditions to swelling-derived network changes, while the proposed additional carbon–carbon crosslinks at 180 °C remained unmeasured [54]. Danielsen et al. surveyed the methods that could settle such questions, and their limits, for networks without an added filler [88].

Antioxidant and plasticiser effects cannot be read cleanly from a fatigue result because the antioxidant contribution couples with ageing time and temperature. In addition, recent work locates fatigue resistance in entanglement density, sacrificial bonds, double networks and chain slip, together with a stiffness–threshold conflict that single networks cannot escape [89,90,91,92,93]. Because the studies cited here measured gels, model elastomers and a glass, their results identify a design axis still to be quantified for vulcanised rubber.

### 7.5. Reversal of Formulation Rankings

Each ranking discussed above depends on its conditions, whether it is the constant-hardness formulation design [81], the matched filler and geometry [32], the HNBR test window [41], or the choice between comparison at constant strain and at constant stress [94]. Change the energy range, temperature, ageing state, load ratio or endpoint, and a ranking can shift or invert. The NR/BR tread series discussed earlier gives a direct example, its blend ranking reversing across a composition-dependent strain transition, although curative level varied with BR content [20].

The twelve-grade carbon-black series shows why such crossings are structural features of a formulation series. Two power-law fits with different exponents intersect at one tearing energy, and in this series, the exponent followed strain amplification while the intercept followed hysteresis, so exponents differed across grades and their fitted curves must cross at some tearing energy. Whether that intersection falls inside the tested range also depends on the formulation, and Tunnicliffe observed exactly such a crossover between carbon-black types, with the performance trade-off it implies [36]. A ranking can also be created or removed by the reduction step alone. In the filler-loading series of Yamabe et al., the trend read from the fitted exponents changed from non-monotonic to monotonic with the fitting range while the compounds stayed the same [27]. Across seven NR/BR formulations, Li et al. found that separately measured self-heating was greatest for the 30/70 blend while the selected pre-cracked curves crossed, and no blend remained best across the reported threshold-to-critical-energy range [72].

Each formulation ranking therefore holds only within the domain where it was observed (geometry and energy convention, load history and thermal state, and endpoint), and the reviewed evidence identifies no filler or polymer as optimal outside that domain.

## 8. Temperature, Ageing and Chemical Environment

### 8.1. Three Routes by Which Temperature Enters

Temperature enters rubber FCG through three physically distinct routes. Direct test temperature, imposed by the chamber, changes the instantaneous viscoelastic and crack tip response. Prior thermal or thermo-oxidative ageing changes the material before loading. Cyclic self-heating creates an evolving, often non-uniform temperature during the measurement while the chamber remains nominally unchanged. A crack growth curve measured at 100 °C is therefore not equivalent to a room-temperature curve obtained after ageing at 100 °C. Table 7 divides the evidence accordingly.

### 8.2. Direct Test Temperature

Higher test temperature was accompanied by faster FCG at a fixed, source-specific tearing-energy level in five of the seven within-study temperature contrasts of Table 7, and by faster crack-contour propagation at fixed strain in a sixth; the NBR contrast of Schieppati et al., described below, shows the same direction [25,53]. In the seventh, growth rose with temperature at low strain but fell at high strain [95]. The magnitude varies with formulation, geometry, energy convention, frequency and the temperature actually attained by the specimen. No universal temperature multiplier therefore follows.

**Table 7:** Evidence for direct test temperature, prior thermal ageing, antioxidant state, cyclic self-heating and chemical environment in rubber FCG. The contrast within each row is the unit of inference. Evidence levels C, P and E are defined in Table 6; “field gap” marks a gap in the literature reviewed here, which was not searched with ozone or fluid terms, rather than a limitation of the listed study alone.

Source	Tested Contrast	Other Conditions	Supported Inference	Boundary	Evidence
Kaang et al. [32]	40 versus 100 °C	N351-filled NR/SBR; pure shear; 1 or 10 Hz	Rate at fixed source-defined *G* rose with temperature, unequally across formulations	Reported approximately tenfold and hundredfold changes: not universal shift factors	C
El Maanaoui and Meier [42]	23, 60 and 90 °C	Carbon-black-filled EPDM/NR; SENT; 10 Hz pulses	Higher temperature increased rate at fixed source-defined tearing energy	SENT relation, coefficient set not fully tabulated: no pooled regression with pure shear	C
Li et al. [96]	23, 40, 60 and 80 °C	Carbon-black-filled HNBR; near-pure shear; variable-amplitude sine, 5 Hz; chamber-controlled elevated temperatures	At constant peak tearing energy, the rate rose nearly tenfold from 23 to 80 °C	Separately fitted exponents fell with temperature; common 1.88 exponent with temperature-dependent prefactor: modelling choice; specimen temperature unreported	C
Béranger et al. [97]	23 versus 120 °C	NBR; source-specific geometry and rate reporting	The within-study direction supports thermal acceleration	Conflicting nm/cycle and nm/min reporting excludes the apparent exact value pair	P
Rublon and Favier [46]	Room temperature, 80 and 100 °C FCG	N330-filled SBR, 2 or 0 phr 6PPD; formulation- and energy-specific programmes	Higher direct test temperature accelerated growth within the tested cases	Long low-energy exposure and one-specimen/two-tip hierarchy limit causal separation	P
Chen et al. [95]	Room temperature, 50 and 80 °C	Non-crystallising S-SBR/BR; 2 Hz; displacement cycled from zero; deviated path; maximum-nominal-strain axis	Growth rose with temperature at low strain and fell at high strain	Path deviation may make *T* = *Wh* inapplicable: no fixed-tearing-energy footing or rate coefficient	P
Schneider et al. [86]	Chamber temperature; strain-based load ratio	Matched 40/60 phr silica- and carbon-black-filled NR; fixed-strain endpoint	Crack-contour propagation rose with chamber temperature, fell with load ratio, tended lower with silica	Fixed-strain endpoint yields no common d*c*/d*N*–*T* coefficient set	P
Narynbek Ulu et al. [41]	Formulations at a fixed 120 °C	Six HNBR blends; crack growth chamber set to 120 °C	Within-programme comparison of high-temperature formulation performance	No 23 → 120 °C or prior-ageing contrast; crack growth curves lack specimen temperature	C (formulation contrast)
Soma et al. [33]	Prior ageing at 100 °C for stated durations	Subsequent source-defined FCG test	Prior ageing changed the later-tested material state	The 1.72× result at *T* = 6300 J m^−2^ and 168 h is not a general multiplier	C
Rublon and Favier [46]	Antioxidant/formulation and exposure history	Low- and high-energy source protocols	Antioxidant-related differences depend on energy and exposure history	Low-energy observations exploratory and correlated within specimen	E
Wang et al. [34]; Shangguan et al. [35]	Variable amplitude with proposed heat build-up	Shared dataset; no specimen thermometry	Self-heating is a plausible explanation requiring direct measurement	2100 J m^−2^ is a model-exclusion boundary, not a measured thermal threshold	E
Liu et al. [31]	Pulse versus sine excitation at 20 Hz	Silica-filled S-SBR; displacement-controlled *R* = 0	Both pulse histories: more surface heat build-up, faster pre-cracked growth than sine	Surface measurement only; separation largest at the shorter excitation fraction; history-specific	C
Li et al. [72]	Self-heating across blend ratio	Seven reinforced NR/BR formulations; separately measured self-heating	Self-heating greatest for the 30/70 blend; selected pre-cracked curves crossed	No blend remained best across the reported threshold-to-critical-energy range	C
Abdelmoniem and Yagimli [98]	Component-scale self-heating	Rotating-shear NR component; surface thermometry	Nearly steady surface temperature coexisted with a still-evolving axial force	Surface steady state does not establish through-section thermal equilibrium; endpoint not pre-cracked FCG	E
Cho et al. [99]	Dry versus swollen state, including SBR swollen in mineral oil	SBR and NBR; dynamic FCG	Swelling changed the observed rate; the source also compared rates at a tearing energy corrected for the fewer chains crossing the fracture plane	One of two dynamic-FCG swelling studies identified here, with Busfield et al. [30]; no cross-study direction or magnitude	C; field gap

Kaang et al. provide the most closely matched paired comparison among the studies reviewed here. NR and SBR compounds containing the same 50 phr of N351 carbon black were tested in pure shear at 40 and 100 °C under fully relaxing loading at 1 or 10 Hz [32]. Across the reported energy range of approximately 300–60,000 J m^−2^, the rate at fixed *G* increased by about one order of magnitude for NR and two for SBR as temperature rose. The measured response contains formulation-specific hysteresis, crack tip deformation and any temperature dependence of the energy calculation, and the approximately tenfold and hundredfold changes are therefore a controlled within-study contrast that does not provide general NR and SBR shift factors. One candidate mechanism is the temperature dependence of SIC, which is governed by crystallisation and melting kinetics [73]. Strain-crystallising and non-crystallising rubbers are therefore not expected to respond to a temperature change in the same way, although none of the reviewed temperature contrasts measured crack tip crystallinity.

El Maanaoui and Meier observed the same direction in carbon-black-filled EPDM and NR SENT experiments at 23, 60 and 90 °C [42]. Their representation retained one exponent and introduced a temperature-dependent prefactor, usefully distinguishing a shift in curve position from a change in slope. However, the article does not tabulate a complete coefficient set, and its SENT relation differs in measurement layer from the pure-shear definition used by Kaang et al. The two support a qualitative thermal trend but not a pooled regression. Li et al. found the same direction in carbon-black-filled HNBR tested at 23, 40, 60 and 80 °C in a near-pure-shear specimen, with a temperature-controlled chamber used at the elevated temperatures. At constant peak tearing energy, the rate rose nearly tenfold from 23 to 80 °C [96]. The separately fitted exponent fell as temperature rose, but the authors described all four temperatures with a common exponent of 1.88 and a temperature-dependent prefactor, a modelling choice of the kind discussed below.

Béranger et al. observed faster NBR FCG at 120 °C than at 23 °C in notched pure-shear tests, a direction supported by their published figure. The rate pair given in their text, however, mixes nm/cycle and nm/min, does not match the plotted locations and is not used here [97].

Rublon and Favier studied N330-filled SBR with either 2 phr or no N-(1,3-dimethylbutyl)-N′-phenyl-*p*-phenylenediamine (6PPD), separating direct test-temperature from prior-ageing experiments [46]. For unaged compounds, crack growth at fixed tearing energy differed by almost a decade between room temperature and 80 °C. Protected and unprotected materials remained similar within the acknowledged scatter at room temperature and 80 °C, whereas the unprotected compound grew faster at 100 °C. The authors proposed that a temperature between 80 and 100 °C may be needed to resolve the antioxidant effect in their system and called for duplication. In a further NBR contrast from one laboratory, Schieppati et al. showed that, in double-edge-notched pure shear at 4 Hz and *R_ε_* = 0.5, stable growth at fixed source-specific *G* rose by nearly two orders of magnitude as chamber temperature increased from 25 to 80 °C [25,53].

The concordant contrasts are heterogeneous in magnitude because an apparent temperature effect can enter the prefactor, the exponent, the accessible regime or the measured thermal state. In the seventh contrast, the crack path of a non-crystallising S-SBR/BR compound grew jagged at 50 °C and branched at 80 °C. Chen et al. judged that this may make *T* = *Wh* inapplicable and attributed the high-strain decrease to path deviation lowering the crack tip driving force [95].

### 8.3. Prior Thermal Ageing and Antioxidants

Thermal ageing can alter crosslink structure, stiffness, molecular mobility, additives and oxidation state before the crack growth test begins, and the later FCG experiment therefore probes a changed material under its own test protocol. Soma et al. made the distinction experimentally. They aged carbon-black-filled NR in circulating air at 70 or 100 °C for 24, 72, 120 or 168 h and then ran pure-shear FCG at room temperature under displacement control, fully relaxing, at 3 Hz over 25–65% tensile strain [33]. The power-law slopes ranged from 1.77 to 2.01. At a tearing energy of 6300 J m^−2^, ageing at 100 °C for 168 h increased the crack growth rate by approximately 1.72 times, whereas the rate was almost unchanged across specimens aged at 70 °C. The 1.72-fold value holds for one compound, one room-temperature FCG protocol, one tearing energy and one ageing condition. Soma et al. connected the response to tensile properties and nuclear magnetic resonance (NMR) measurements of molecular mobility, which supports a material-state interpretation without identifying a unique causal path. Changes in stiffness also modify the strain-energy density associated with a prescribed strain.

Rublon and Favier exposed their protected and unprotected SBR compounds to air at 80 °C for 16, 168 or 840 h [46]. At room-temperature FCG, differences after 16 and 168 h were not resolved beyond the scatter of the method. After 840 h, crack growth resistance decreased, particularly for the unprotected compound, and at higher tearing energies, and a similar pattern appeared when FCG was tested at 80 °C. In contrast, Béranger et al. found comparatively small ageing-related changes after exposure at 80 °C for up to 21 days, relative to the pronounced direct test-temperature effect in their NBR/HNBR systems, the aged series being tested at 120 °C [97]. Direct heating and prior ageing therefore need not produce comparable shifts even when both are expressed through FCG curves.

The antioxidant contrast of Rublon and Favier also changes with the energy range. At 80 °C and 100 J m^−2^, one specimen containing 2 phr 6PPD gave right- and left-crack rates of 0.00178 and 0.0128 nm/cycle, while one unprotected specimen gave 0.65 and 0.18 nm/cycle [46]. The two crack tips within each specimen are correlated subsamples. Because each low-energy test lasted about 10 days at 80 °C, concurrent exposure is inseparable from the measured rate. The result is exploratory and should not be averaged into specimen-level statistics. On the other hand, at higher energies and at room temperature or 80 °C, protected and unprotected compounds were often indistinguishable within the reported scatter until the longest exposure. The role of 6PPD thus depends on whether the question concerns low-energy growth, high-energy growth, direct elevated-temperature testing or resistance retained after ageing. More generally, a formulation contrast observed after thermal exposure is best described first at the response level (rate, energy range, temperature and duration), with a chemical mechanism assigned only on direct measurements of additive depletion, network evolution or oxidation state.

### 8.4. Self-Heating

Cyclic self-heating develops during the test and couples rate, strain amplitude, specimen thickness, convection and hysteresis, and the temperature may therefore evolve with cycle number and differ between surface, bulk and crack tip region. It can alter the operative crack growth response and, at the same time, invalidate the assumption that a curve was measured at a fixed thermal state. In the Shangguan/Wang dataset, the rate accelerated markedly above the upper bound of the fitted domain, which the authors attributed partly to temperature accumulation [34,35]. Without thermometry, that reading cannot be separated from strain-rate, geometric, regime and load-history alternatives.

A within-study route can indicate self-heating without a thermometer, as Yamabe et al. showed. They ran their FCG tests in air at a nominal 23 °C, 5 Hz and a strain ratio of 0.1, and separately measured static crack growth at several temperatures on the same material. Replotted as growth per unit time against growth per cycle, the time-based rates of the room-temperature fatigue tests matched those of the static tests at 40 °C, which the authors took to suggest self-heating [27]. Although this 40 °C value was inferred and not measured, it shows that a curve labelled “room temperature” can carry an operative thermal state well above the set point.

One direct measurement and one component observation indicate that a controlled or steady surface temperature does not fix the temperature through the section. Gehrmann et al. used forced convection to hold the specimen surface near room temperature during fatigue experiments and measured a bulk–surface difference of up to 8 °C [100], on a fatigue-life endpoint. Kocjan et al. treated thermal state as part of the transformation between room-temperature FCG, crack-nucleation fatigue and an isothermal three-dimensional dumbbell response [52]. At the component scale, Abdelmoniem and Yagimli found on a rotating-shear NR component that the surface temperature appeared nearly steady while the axial force continued to evolve, and a surface steady state therefore did not establish through-section thermal equilibrium [98]. Where temperature is not measured, suspected self-heating is best treated in the uncertainty budget or the exclusion criteria instead of being absorbed into a material parameter.

### 8.5. Chemical Environment

In the literature reviewed here, which was not searched with ozone or fluid terms (Appendix S1), the chemical environment entered dynamic crack growth in a few studies: Lake and Lindley examined ozone and oxygen [16]; Lake compared growth in vacuum and in air [58]; Rublon and Favier compared antioxidant contents during growth in air at elevated temperature [46]; Busfield et al. [30] and Cho et al. measured dynamic FCG in swollen elastomers; and Legorju-Jago and Bathias compared crack growth in air, water and dry nitrogen [57]. Cho et al. compared dry and swollen SBR and NBR, including SBR swollen in mineral oil, and showed swelling changing the observed rate and requiring a correction to the tearing energy for the fewer chains crossing the fracture plane [99]. For one SBR compound, the nominal comparison reached an increase of up to about two orders of magnitude, while the source-specific $\lambda_{s}^{2}$ correction to tearing energy removed part of the apparent separation. A separate non-cyclic ozone-threshold case used prestrained NR with 50 phr N550 and 1 phr 6PPD and gave a maximum estimated ozone threshold *T*_z_ = 2.12 J m^−2^ at 50 pphm and 30 °C against a mechanical *T*_0_ = 56.88 J m^−2^ from an Intrinsic Strength Analyser (ISA) cutting measurement, with an assumed 0.10 mm interacting-crack depth that prevents a universal correction or cyclic law [101]. The chemical environment remains a research gap, with a weaker evidence base than temperature and loading history (Table S2). Further synthesis requires primary studies reporting fluid concentration, exposure duration, temperature, geometry, energy convention, swelling correction and load minimum together.

### 8.6. Implications for Temperature Correction

The evidence in this section argues against a single horizontal “temperature correction” for rubber FCG because the magnitude of a direct temperature effect does not transfer between test methods, and prior ageing and self-heating enter through separate routes. Instead, a thermal-state record is proposed for every rate curve (direct test temperature, prior exposure history, actual specimen temperature, frequency and waveform, and atmosphere or fluid). Only curves that share this record, together with the measurement variables of Section 4 and Section 5, are then pooled directly. A broader dataset may instead carry temperature as an explicit covariate, provided geometry, energy convention, load history and dependence structure stay in the model. Even then, a temperature-dependent prefactor can summarise a controlled dataset without demonstrating one molecular activation process. Likewise, a change in fitted exponent may reflect a regime transition, thermal gradients, formulation-specific dissipation, the temperature dependence of SIC or the fitted range.

## 9. From Coupon Data to Component Life

**Figure 8:**
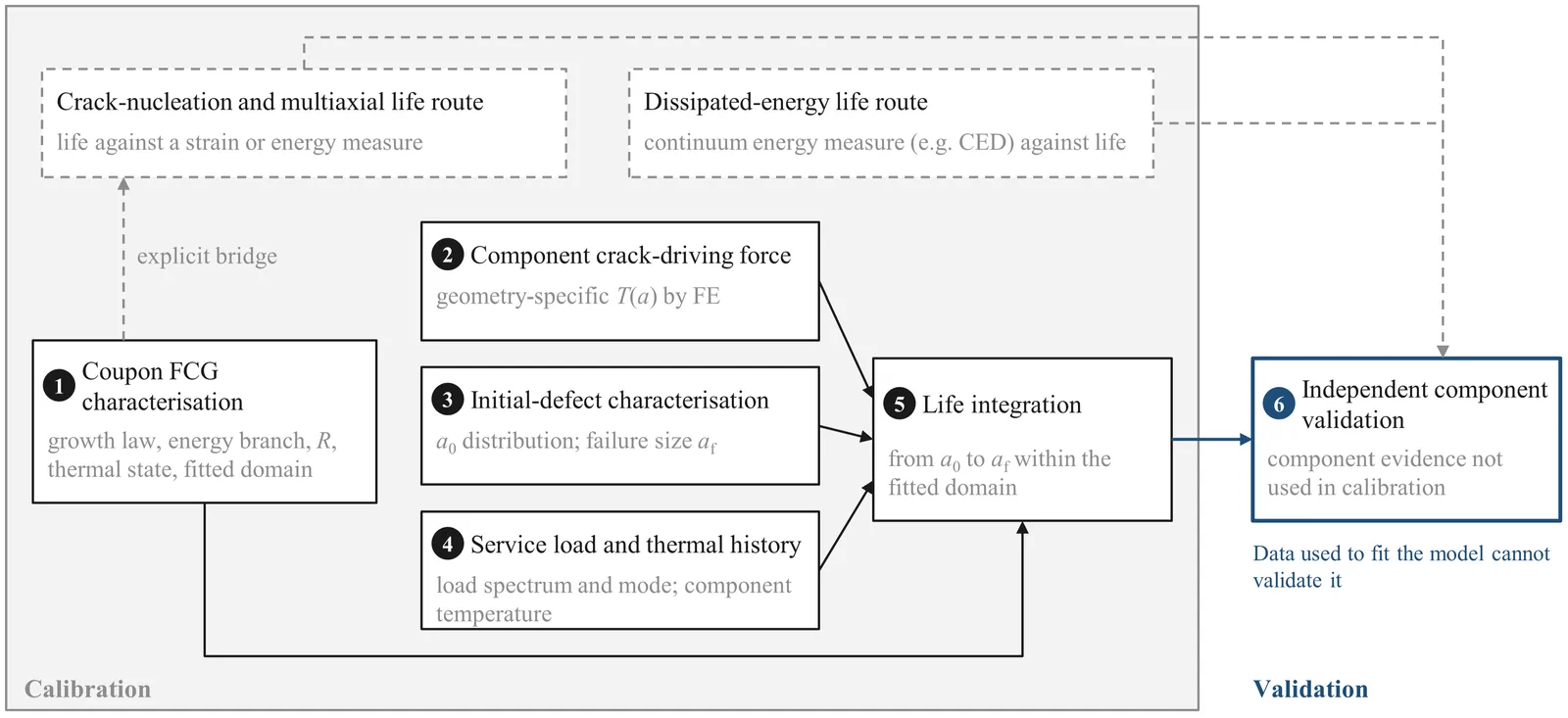
Workflow from coupon fatigue crack growth data to a component life; schematic drawn by the authors. Stages 1–5 build and calibrate the prediction inside the shaded calibration boundary; stage 6, comparison with component evidence not used in calibration, lies outside it. The dashed boxes are the crack-nucleation and multiaxial route, and the dissipated-energy route. Both substitute life relations calibrated on their own endpoints for the crack growth law and its integration and lead to the same independent validation; neither provides coefficients transferable to a d*c*/d*N*–*T* law (Table 8). Coupon crack growth data can enter the nucleation route through an explicit bridge that states geometry, conversion and temperature, as demonstrated by Kocjan et al. [52] (Section 9.3). CED, cracking-energy density; FE, finite element.

A component life calculation built on a coupon crack growth law proceeds through six stages (Figure 8). The first five stages build and calibrate the prediction, whereas only the sixth, a comparison with component evidence not used in that calibration, validates it. A model fitted to a dataset and then reported to agree with the same dataset has therefore been demonstrated but not validated.

### 9.1. Integrating a Crack Growth Law into a Life

A crack growth law characterised under the controlled conditions discussed above yields a fatigue life only when it is integrated from an initial crack size *a*_0_ to a failure size *a*_f_. The integration requires a geometry-specific relation between tearing energy and crack length, *T*(*a*). In a pure-shear coupon the driving force is approximately independent of crack length over the admissible window, which is why the coupon is convenient. In a component, the tearing energy usually rises or falls as the crack extends, so the same material law produces a different life once the component *T*(*a*) is inserted. Table 8 maps the principal transfer routes and their assumptions.

The integration is bounded at both ends by the validity range of the growth law. A fit restricted below a stated cut-off, such as the 2100 J m^−2^ bound of the Shangguan/Wang dataset, cannot be integrated through higher energies without separate justification. At the other end, a power law fitted over a given range carries no information about the threshold region below it. Integrating it down to arbitrarily small tearing energies returns a finite life at every load level, which is inconsistent with the existence of a threshold of the kind Lake and Lindley identified. The fitted interval therefore sets the lower limit of the integration, and extending it downwards requires separate threshold data.

### 9.2. Initial Defects and Failure Criteria

The initial crack size is a consequential and under-characterised input in the reviewed defect-controlled integrations. It is a distribution that depends on material, process and inspection, and a single fitting constant cannot represent it. Li et al. inferred, for an NR component under relaxing loading, a crack-precursor size of about 20–60 µm with a mean of 35.7 µm at *R* = 0, integrating a Thomas growth law to a failure crack size *a*_f_ = 5.00 mm [59]. They attributed the scatter in *a*_0_ to contaminants, voids, imperfectly dispersed ingredients and filler agglomerates (the formulation and processing variables discussed above). Meier et al. approached that population experimentally, using glass spheres as model flaws in EPDM and computed tomography for particle-size distributions and interpreting particles above 50 µm as nucleation sites. They cautioned, however, that the tomographic size distribution cannot be taken as absolute [104].

In the Shangguan/Wang dataset family, dumbbell lives were computed for initial flaw sizes of 20, 40 and 60 µm, and for the 40 µm size that best matched the measured lives, agreement within a factor of two was reported across the load cases [34,35]. Because propagation life varies as $a_{0}^{1-\beta }$ for a small flaw whose tearing energy scales linearly with its size, the fitted *β* = 2.33362 makes the 20–60 µm range alone span a factor of about 4.3 in predicted life, comparable to the full width of the reported factor-of-two band. Because that size was chosen as the best match to the measured lives, the agreement is a calibration result rather than an independent validation (Figure 8). The failure size *a*_f_ is likewise a choice but a bounded one because unstable tearing intervenes once the geometry-specific *T*(*a*) reaches the critical tearing energy of that material state.

The load ratio also modifies the precursor and propagation description. Legorju-Jago and Bathias provided a direct FCG contrast, whereas Li et al. inferred an *R*-dependent law from S–N life data. Together, they show that a life model calibrated for a relaxing cycle transfers to a non-relaxing duty only through an endpoint-compatible *R* treatment [57,59].

### 9.3. Crack Growth, Crack Nucleation and Dissipated Energy

Two modelling routes coexist in component fatigue, pre-cracked crack growth described by d*c*/d*N*–*T*, and crack nucleation described by a life against a strain or energy measure. Depending on material, geometry and flaw population, the nucleation stage can occupy a large fraction of the observed life, and the two therefore answer different design questions even for the same part. Kocjan et al. demonstrated an explicit bridge, converting a planar-tension crack growth measurement into a crack-nucleation-fatigue (CNF) prediction for uniaxial and three-dimensional dumbbell geometries. For the fatigue tests in their study, if conducted serially, they estimated about 20 h for the crack growth route and about 1600 h for the nucleation route [52]. The bridge is valuable because it makes geometry, conversion and required temperature explicit, and it does not imply that a nucleation life can be read off a d*c*/d*N*–*T* curve. Luo et al. kept the two routes labelled when predicting the life of cord/rubber laminates [66].

Multiaxial evidence forms a ladder of distinct endpoints instead of a pool of FCG data. Poisson et al. treated combined tension–torsion failure life, Saintier et al. defined initiation by a 1 mm crack, and Verron and Andriyana modelled nucleation or complete-failure life [105,106,107]. Dissipated-energy-density predictors, including hysteresis-based cracking-energy density (CED), bulk-versus-surface energy treatments and component life models [100,108,109], relate a continuum energy measure to a life, usually complete fracture. They carry their own *R* and thermal boundaries and form alternative, endpoint-specific routes to a life beside the d*c*/d*N*–*T* route (Figure 8).

### 9.4. Geometric, Thermal and Load Transfer to Components

A component life calculation composes separate inputs (a coupon crack growth or nucleation law, a geometry-specific *T*(*a*) from a stress analysis, an initial-defect distribution, a thermal history and a load history). Transfer errors originate wherever one of these is left implicit. Finite-element (FE) analysis supplies the geometry term. Pidaparti et al., for example, computed tearing energy for cracked rubber components in two and three dimensions using a virtual-crack-extension method with a maximum-tearing-energy direction criterion [102]. The thermal term should be the actual component temperature because self-heating may shift or confound the operative response. In a separate study, Luo et al. combined monotonic critical energies measured from −40 to 70 °C with an FCG relation measured only at 23 °C, and their higher-temperature rates were therefore model outputs and not measurements [110].

The load term must respect both the load spectrum and the loading mode of the service duty. Harbour et al. tested variable-amplitude, multiaxial histories on NR and SBR and examined Miner’s linear-damage rule for predicting their lives [111]. Demassieux et al. provided a direct mode-III FCG example using a circumferentially cut bonded disk under cyclic torsion with a small axial extension to limit crack-face abrasion; their fracture-energy construction combined an analytical relation with global stored-energy FE checks and remained sensitive to boundary conditions [112]. Gosar et al. showed the combination in practice, coupling a multiaxial energy-based damage model with a mean-stress correction and comparing its predictions with tests on a commercial-vehicle air spring [67].

Three cases delimit the transfer demonstrated so far. A crack-nucleation life curve measured on a three-dimensional dumbbell was transformed, through a strain-energy measure and a thermal correction, into a prediction that was compared, like the prediction from planar-tension FCG, with measured uniaxial lives [52]. A crack growth route applied to a cord/rubber laminate overpredicted life because interfacial degradation was not represented. This was a failure-mode mismatch and did not indicate a defect of the growth law [66]. Finally, an air-spring FE model carried a fatigue-damage field that located the critical region for a component-scale calculation [67]. The three cases remain separate examples of individual transfer steps (Table 5).

### 9.5. Validation and Uncertainty

Uncertainty should be propagated through the calculation instead of being summarised by a single ratio. The 20–60 µm *a*_0_ range discussed above materially changes defect-controlled life integrations. Belkhiria et al. propagated dispersion in Wöhler material parameters for a cracking-energy-density life endpoint using Monte Carlo simulation and FE analysis [103]. This is uncertainty-aware life modelling, though not a treatment of a measured initial-defect distribution. A transfer report should identify each calibration input of Figure 8 separately, including parameter covariance, so that a coupon relation becomes a testable component prediction.

### 9.6. Illustrative Case: Heavy-Duty Tracked-Vehicle Components

Heavy-duty rubber components such as track pads and track shoes combine many of the difficulties discussed above. Within a duty cycle, they may experience repeated compression and shear, impact, cutting and chipping, abrasion, self-heating, environmental exposure and process-related flaws. Track, road-wheel and terrain interactions are expected to produce multiaxial, variable-amplitude local histories. The available application evidence, a single technical report, directly documents wear, cyclic compression, heat build-up, tear and crack-related damage [113]. Young noted the laboratory–service gap early, finding strain-rate effects on strain energy small at a tyre-service-equivalent 40 Hz but large at typical laboratory frequencies of ≤5 Hz, and dependent on polymer and temperature [114].

**Figure 9:**
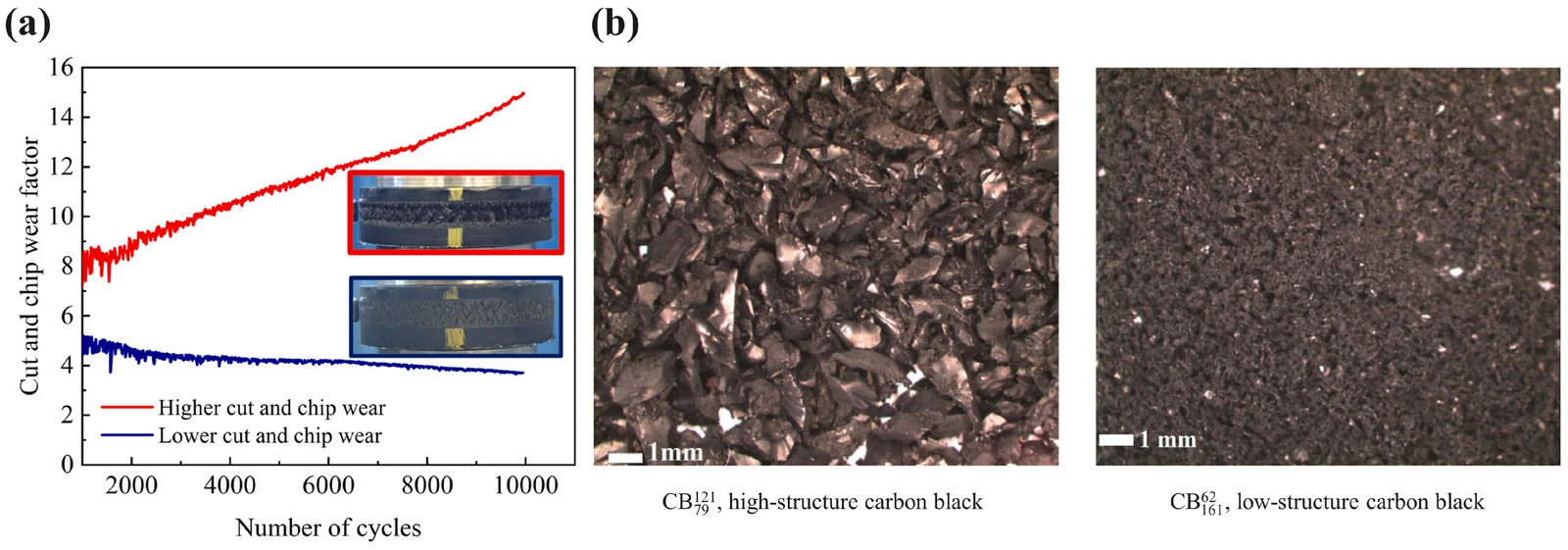
Cut-and-chip observations. (**a**) Cut-and-chip wear factor, calculated in situ from changes in the measured range of the shear force, against number of cycles for two test wheels with higher and lower cut-and-chip wear; the insets show the corresponding wheel wear surfaces. (**b**) Debris morphology at 200 N: optical images of debris from the high-structure CB^121^_79_ and low-structure CB^62^_161_ carbon-black (CB) compounds; superscripts and subscripts denote structure as compressed oil absorption number (COAN) and surface area as statistical thickness surface area (STSA), respectively. Adapted from Ref. [115] (source Figures 3 and 15A,B; © 2024 The Authors, published by Elsevier B.V.) under a CC BY 4.0 licence (https://creativecommons.org/licenses/by/4.0/ (accessed on 7 October 2026)); panel (**a**) reproduces source Figure 3 without modification, and in panel (**b**), the debris images were cropped and rearranged, and their labels reformatted, with image content, borders and scale bars unchanged.

The track-pad case separates the damage modes that an FCG model can address from those governed by wear, cut-and-chip or impact. Current evidence supplies material screening for NR and high-temperature HNBR compounds, comparability criteria and a method for integrating a growth law into a life. Between a laboratory d*c*/d*N*–*T* curve and a validated track-pad life lie the geometry, defect and thermal inputs and the multiaxial, variable-amplitude load inputs that the available track evidence does not report. Kyei-Manu et al. resolved distinct low-force and high-force cut-and-chip regimes [115], and Figure 9 shows the in situ wear factor and debris morphology from that study. Cut-and-chip wear is an endpoint that lies outside a cyclic d*c*/d*N*–*T* fit and its crack growth rankings.

## 10. Research Priorities

Three experiments are proposed, each directly testing a comparison item or evidence gap that limited the comparisons examined here. First, a controlled round-robin on one compound should compare pure-shear, SENT and DCB geometries under one energy-accounting convention. With at least one geometry repeated across laboratories, the round-robin directly tests the completeness of the checklist. Second, a factorial temperature–load-ratio–frequency programme should combine direct specimen thermometry with strain-crystallising and non-crystallising reference compounds. Third, matched environmental conditioning followed by one FCG protocol should separate material-state change from test-state change. Table S2 gives the response variables, replication, crack resolution and comparison strategy for these three experiments and specifies four further priorities (track-representative spectra, defect-population transfer, observation of endpoint transitions and open raw data).

## 11. Conclusions

Rubber FCG parameters depend on how they were measured as well as on the compound tested. Once the test conditions behind each result were made explicit, apparently general conclusions resolved into controlled material contrasts, bundled changes or unresolved comparisons, as the six cases in Table 5 illustrate.

The two-stage, seven-item checklist (Table 3) therefore admits a quantitative comparison only when the endpoint matches; coefficients share their energy definition; data reduction is matched by re-deriving rates from the raw crack records; and each of the geometry, load-minimum, rate-and-history and thermal items either matches or has had its effect measured by a within-study contrast over the relevant range on the same base polymer, the comparison stating that bound. Otherwise, the unresolved item restricts the result to qualitative or within-study use. The minimum reporting set in eight domains (Table 4) records what the checklist needs, and the round-robin of Section 10 would test the checklist’s completeness.

When read against the checklist, the backbone, filler, network and temperature effects hold within matched endpoints and test conditions (Table 6 and Table 7), and the reviewed evidence does not support a state-independent formulation ranking. When formulations differ in exponent, their curves intersect, within or beyond the tested range, and a ranking therefore holds only on the side of the crossing where it was measured. The lower exponent of strain-crystallising NR relative to non-crystallising SBR is an ordering seen in most exponents reported for both, none from a study that isolated the backbone, whereas the exponent values of both rubbers shift with the test conditions between programmes. Prior ageing needs to be indexed by its own exposure history, separately from test temperature. The load minimum is mechanistically active but defined in several different ways among the studies compared here, and the chemical environment remains a research gap.

Component prediction further requires a geometry-specific crack-driving history, initial and final defect criteria, operative load and temperature histories, uncertainty propagation and validation independent of calibration (Figure 8). For heavy-duty tracked-vehicle components, the available track evidence does not yet supply these inputs.

## Figures and Tables

**Table 1:** Coverage of the comparison items of this review in representative earlier reviews of rubber fatigue. Coverage was coded from the full text of each review as developed (central and treated in depth), covered (treated but not the organising principle), brief (mentioned or contextual) or not addressed (no substantive treatment found); intermediate cells are coded as a range, for example covered to developed.

Review	Endpoint Separation	Specimens, Energy Definition and Rate Measurement	Loading History and Thermal State	Treatment of Cross-Study Comparability	Coupon-to-Component Transfer
Mars and Fatemi, 2002 [1]	Developed: nucleation and crack growth as separate routes	Covered: fracture mechanics basis, tearing energy and growth laws	Brief: deferred to a companion survey	Not addressed	Developed: flaw-growth integration and engineering examples
Tee et al., 2018 [2]	Developed: nucleation, propagation and fractography	Developed: specimens, tearing energy and growth laws	Developed, as influencing factors	Brief: factors catalogued	Covered: life predictors and selected applications
Qiu et al., 2022 [5]	Developed: initiation and propagation methods	Covered: specimen classes, tearing energy and propagation laws	Developed, as influencing factors	Brief	Covered: prediction methods
Gehling et al., 2023 [3]	Covered to developed: FCG central, initiation distinguished	Developed: methods, specimens, procedure and evaluation	Developed: service and test conditions, hysteretic heating	Covered: test variables discussed narratively	Covered: component durability as motivation
Wang et al., 2025 [4]	Developed: nucleation, crack growth and probabilistic routes	Developed: energy release rate, specimens and test approaches	Covered to developed: load ratio, amplitude history, frequency and temperature	Covered: specimen choice and test variables recognised	Developed: applications and the material-to-component gap
Głowacka et al., 2025 [6]	Covered, within composite fatigue	Covered: selected crack growth examples	Covered: frequency, hysteretic heating and thermal ageing	Brief	Covered to developed: tyres and cord–rubber systems
Ning et al., 2026 [9]	Developed: initiation and propagation as two approaches	Covered: energy release rate defined, energy branch not specified	Covered: load ratio, frequency and temperature as factors	Not addressed	Brief: Paris law for life prediction
This review	Endpoint check precedes all other items (endpoints in Table 2; item 1 of Table 3)	Geometry and constraint, energy definition and branch, data reduction and replicates (items 2, 3 and 7)	Load minimum, rate and history, thermal state (items 4–6)	Two-stage, seven-item checklist: endpoint a full gate, energy definition a gate for coefficients, geometry, load-minimum, rate-and-history and thermal items, where not matched, admitting only a comparison bounded by a within-study contrast measured on the same base polymer, and data reduction matched by re-deriving rates, with resolution and replicates matched as recorded	Transfer routes with their required evidence and validation basis (Section 9)

**Table 4:** Minimum reporting set for assessing the compatibility and reuse of rubber fatigue crack growth data. An unreported item is treated as unresolved. The last column names test standards whose published scope bears on the domain: ISO 27727, fatigue crack growth rate in pure shear [37]; ASTM D813, flex crack growth of a pierced specimen [63]; ASTM D4482, extension cycling fatigue life [64]; ASTM D624, tear strength [65]. A standard fixes the procedure of its own method, whereas the minimum record applies whichever method is used.

Domain	Minimum Record	Compatibility Question	Consequence When Unresolved	Test Standards (Published Scope)
Material state	Polymer grade and blend; filler grade/loading; additives; cure; batch; ageing/storage state	Is the material state specified and controlled?	Rank materials qualitatively only	—
Specimen and crack	Geometry, dimensions, constraint/mode; notch or pre-crack preparation; crack orientation; admissible crack-length window	Are the crack tip fields compatible?	Do not pool on shared units alone	ISO 27727; ASTM D813; ASTM D4482; ASTM D624
Driver and energy accounting	Definition of *W*, *T*, *G*, Δ*G* or *J*; loading/unloading branch; geometry relation or field method; valid range	Are the drivers built from equivalent quantities?	Keep coefficients branch- and geometry-specific	ISO 27727
Cycle definition	Control quantity; minimum, maximum and definition of *R*; waveform, frequency, dwell, sequence, preconditioning	Are minimum states and histories compatible?	Treat the loading contrast as bundled	ISO 27727; ASTM D813; ASTM D4482
Thermal and environmental state	Chamber condition; surface, bulk or local thermometry; steady-state criterion; prior exposure, equilibration	Is the specimen state known rather than inferred from a set point?	Do not attribute differences to material or frequency alone	—
Observation and reduction	Crack/path definition; spatial and temporal resolution; calibration; transient exclusion; smoothing, fitting, differentiation	Can rate and driver be reconstructed from the observations?	Do not reuse absolute rates without a documented reduction	—
Replicate independence and data origin	Batch–specimen–crack front–segment–derived-point hierarchy; shared dataset or model ancestry	What is the independent evidence unit?	Count shared/correlated data once at the right level	—
Endpoint, transfer and validation	Endpoint and detection/failure criteria; *a*_0_, *a*_f_; calibration data; independent validation; uncertainty	Does the evidence answer the same question and support the transfer?	Keep initiation, propagation, life, wear and model outputs separate without a validated bridge	ISO 27727 (FCG rate); ASTM D813 (flex crack growth); ASTM D4482 (fatigue life); ASTM D624 (tear strength)

**Table 5:** Cases in which an unresolved comparison item changes the interpretation of rubber fatigue evidence. The final column states the strongest conclusion supported by the endpoint, test conditions and independence of the underlying data.

Evidence Case	Apparent Conclusion	Unresolved or Conflicting Item	Supported Conclusion
South et al. [26], DCB versus SEN	The rate difference is a universal plane-strain correction	Geometry, constraint, driver relation and frequency changed together	A bundled within-programme geometry–constraint–frequency contrast; no universal conversion factor
Tunnicliffe [36], loading- and unloading-derived tearing energy	One exponent and one intercept can be selected as material constants	Loading and unloading branches produced separately fitted parameter pairs	Each coefficient pair remains tied to its energy branch and fitted range
Shangguan et al. [35] and Wang et al. [34]	Two publications independently confirm the variable-amplitude relation	Specimens, crack histories, processing, coefficients and validation data overlap	One dataset family; the relation is usable only within its stated material, history and *T*_max_ < 2100 J m^−2^ fit domain
Kaang et al. [32] and other direct-temperature studies	A common temperature multiplier can shift rubber FCG curves	Formulation, geometry, energy definition, frequency and specimen temperature are not aligned across studies	Concordant in direction except one high-strain reversal; condition-specific within-study acceleration; no pooled multiplier
Rublon and Favier [46], low-energy antioxidant comparison	Two crack tips provide replicate statistics and isolate antioxidant content	Two correlated crack tips came from one specimen per formulation, with concurrent exposure during the low-energy test	Exploratory formulation- and exposure-specific contrast; no specimen-level mean or unconfounded antioxidant effect
Kocjan et al. [52], Luo et al. [66] and Gosar et al. [67]	Coupon and component cases form one validated transfer sequence	Endpoints, geometry transformations, failure modes, calibration and validation bases differ	Separate bridge, mismatch and component-application examples; no pooled validation or universal transfer rule

**Table 6:** Formulation levers and their within-study effects on rubber fatigue crack growth. Each entry holds only under the listed conditions. Evidence: C, controlled within-study contrast (one programme, one variable changed, others held); P, partially controlled (two or more variables changed together, or one variable changed without a common source-defined driving force or consistent units); E, exploratory (an observation that does not isolate a contrast, such as correlated or concurrent exposures or a secondary citation). Unreported conditions leave the level unchanged.

Lever	Within-Study Effect	Conditions Under Which It Was Observed	Where It Reverses or Remains Unresolved	Refs.	Evidence
Backbone: strain-crystallising NR versus non-crystallising SBR	Lower NR exponent (tabulated 2.08–2.26 versus 3.78–4.21); from 40 to 100 °C, rate at fixed *G* rose by about one order of magnitude (NR) and two (SBR)	N351-filled; one pure-shear programme; *G* = *Uh*_0_; 1 and 10 Hz	Backbone not isolated from formulation; SIC not measured	[32]	P
Carbon-black grade at constant hardness	Exponent 1.54–3.32 (unloading branch), falling as calculated strain amplification rose (2.93–3.90); intercept moved with hysteresis; curves of grades with different exponents cross	Twelve grades at 39–108 phr in one NR matrix; plane-strain pure shear	Grade and loading vary together; coefficients remain branch-paired	[36]	P
Carbon-black particle size and structure	Smaller-particle, higher-structure grades slower; second series: high-structure up to two orders of magnitude slower than low-structure below a step near 2500 J m^−2^	50 phr in one NR matrix; second series in pure shear at 4 and 2.5 Hz, unloading-branch *T*	Separation narrowed above about 25% strain, especially at 70 and 100 °C; compounds converged above the step; whether its position is a material property is open	[62,70]	P
Filler loading	Exponent trend non-monotonic with all data fitted, monotonic with high-energy data excluded	Carbon-black- and silica-filled SBR, 5–25 vol%; pure shear	Apparent minimum near 25 phr attributed by the authors to the fitting range	[27]	C
Silica with TESPT silane versus carbon black	Threshold near 500 versus 170 N m^−1^, roughly threefold at comparable hysteresis ratios, at about 20 vol% and above	One SBR matrix; loading branch; 5 Hz; strain ratio 0.1; 20 µm resolution after 2 × 10^6^ cycles; specimen not strictly pure shear, finite-element (FE)-validated central band	Filler systems as compounded, not filler surfaces; hybrid networks unresolved	[27]	P
Cure system at matched crosslink density	Unfilled: indistinguishable; 40 phr HAF: shared low-strain slope 1.26, at high strain 2.45 (semi-EV) and 3.43 (EV)	NR; strain control at 15 Hz over 23–85% strain; before ageing	Ordering changes after ageing	[69]	C
ACN content and hydrogenation in HNBR	A36H96 best; at 50 J m^−2^, about 7 nm/cycle for A36H91 against about 0.2 nm/cycle for the other two blends	70 phr N772; peroxide cure; 120 °C; 50–1000 J m^−2^	Restricted to the tested window and compositions	[41]	C
Curative level and blooming	Surface state and cyclic growth changed with curative level	Silanised-silica-filled NR; *R* = 0; 1.4 Hz	Absolute rates available only as plotted curves	[71]	P
NR/BR blend ratio	Ranking reversed at a composition-dependent transition, roughly 20–35% strain; separate series: pre-cracked curves crossed	Tread blends; seven reinforced NR/BR formulations	No blend best across the reported range; curative level varied with BR content in the tread series	[20,72]	P [20]; C [72]
Antioxidant 6PPD	Lower rates with 2 phr 6PPD at 80 °C and 100 J m^−2^; unprotected compound faster at 100 °C	N330-filled SBR; low-energy tests lasting about 10 days	One specimen per formulation, two correlated crack tips and concurrent exposure: exploratory only	[46]	E

**Table 8:** Routes from coupon fatigue measurements to fatigue or component life. Reported agreement is classified by its actual calibration and validation basis.

Route and Example	Endpoint Transformation	Required Evidence	Supported Use	Boundary
Pre-cracked FCG integration; Shangguan et al. [35]/Wang et al. [34]	d*c*/d*N*[*T*(*a*), state] integrated from *a*_0_ to *a*_f_	Compatible growth law; geometry-specific *T*(*a*); defect and failure definitions; full load/thermal history	Conditional propagation-life calculation within the fitted domain	Shared dataset counts once; do not integrate beyond *T*_max_ < 2100 J m^−2^ without new support
Bridge from FCG to crack-nucleation fatigue (CNF); Kocjan et al. [52]	Planar-tension FCG related to uniaxial/3D dumbbell life	Source-specific geometry and thermal–finite-element (FE) conversion; compound calibration; endpoint definitions	Demonstrates an explicit bridge and its experimental-efficiency motivation	Not direct endpoint equivalence or a transferable coefficient
S–N-informed defect model; Li et al. [59]	Life data inform an *R*-dependent propagation model	Stated inference route for *a*_0_, *a*_f_ and non-relaxing response	Illustrates defect-size sensitivity and load-ratio dependence	Inferred mean defect size is not a universal rubber flaw distribution
Component-specific crack or damage model; Luo et al. [66], Gosar et al. [67]	Laminate or component geometry enters a route-specific life/damage model	Failure mode, constituent response, FE/driver assumptions and independent component evidence	Shows coexistence of route-specific component models and possible mismatch	Do not merge nucleation, interface degradation and pre-cracked FCG endpoints
Geometry/FE driver route; Pidaparti et al. [102]	Crack geometry and path determine the component driver	Mesh/convergence, path or mode criterion, compatible material law and histories	Supplies a geometry-specific energy/direction calculation	Geometry evidence alone is not component-life validation
Probabilistic or dissipated-energy life; Belkhiria et al. [103] and related studies	Wöhler/cracking-energy-density (CED) or probabilistic damage relation coupled to FE	Endpoint-specific calibration; uncertainty; component loads; external validation	Supports uncertainty-aware life modelling in its declared endpoint	Do not convert CED coefficients to a tearing-energy-based d*c*/d*N* law

## Data Availability

No new data were created or analyzed in this study. Data sharing is not applicable to this article.

## Supplementary Materials

The following supporting information can be downloaded at https://www.mdpi.com/article/10.3390/polym18202476/s1, Table S1: Full measurement-layer matrix of core rubber FCG studies; Table S2: Prioritised research agenda for comparable and transferable rubber-fatigue evidence; Appendix S1: Archived query record, update searches and targeted-retrieval stream.

## Abbreviations

The following abbreviations are used in this manuscript:

6PPD	N-(1,3-dimethylbutyl)-N′-phenyl-*p*-phenylenediamine
ACN	acrylonitrile
AI	artificial intelligence
BR	butadiene rubber
CB	carbon black
CCD	charge-coupled device
CED	cracking-energy density
CNF	crack-nucleation fatigue
COAN	compressed oil absorption number
DCB	double cantilever beam
DIC	digital image correlation
EPDM	ethylene–propylene–diene rubber
EV	efficient vulcanisation; semi-EV—semi-efficient vulcanisation
FCG	fatigue crack growth
FE	finite element
HAF	high-abrasion-furnace carbon black
HNBR	hydrogenated acrylonitrile–butadiene rubber
IR	synthetic polyisoprene rubber
ISA	Intrinsic Strength Analyser
N330, N351, N550, N650, N772, N990	carbon-black grade designations
NBR	acrylonitrile–butadiene rubber
NMR	nuclear magnetic resonance
NR	natural rubber
PDMS	poly(dimethylsiloxane)
PMMA	poly(methyl methacrylate)
phr	parts per hundred parts of rubber, by mass
pphm	parts per hundred million
SBR	styrene–butadiene rubber; S-SBR—solution-polymerised SBR
SEN	single-edge-notched
SENT	single-edge-notched tension
SIC	strain-induced crystallisation
S–N	applied stress or strain against the number of cycles to failure
STSA	statistical thickness surface area
TESPT	bis[3-(triethoxysilyl)propyl] tetrasulfide (Si69)
WAXD	wide-angle X-ray diffraction
