# Supplementary material for: Fatigue Crack Growth in Rubber: Comparability of Test Results, Material Effects and Transfer to Component Life—A Critical Review
Source: Polymers (Basel). 2026 Oct 10;18(20):2476. doi: 10.3390/polym18202476 (PMC13709220; doi:10.3390/polym18202476)
Supplement: Supplementary file 1 [file polymers-18-02476-s001.zip › polymers-4624389-supplementary.pdf]

Fatigue Crack Growth in Rubber: Comparability of Test Results, Material Effects and Transfer to Component Life—A Critical Review

Supplementary Materials

Jeong-Hwan Yoon*, Kyoung-Ho Shin, Ye-Won Park and Yeon-Jee Cho

Sustainable Materials R & D Center, Korea Automotive Technology Institute, 303 Pungse-ro, Pungse-myeon, Dongnam-gu, Cheonan-si, Chungnam 31214, Republic of Korea

* Correspondence: jhyoon@katech.re.kr

Contents

Table S1. Full measurement-layer matrix of core rubber FCG studies

Table S2. Prioritised research agenda for comparable and transferable rubber-fatigue evidence

Appendix S1. Archived query record, update searches and targeted-retrieval stream

Reference numbers are those of the main article. The entries cited in this Supplementary Materials are listed in full at the end of this file.

Abbreviations and symbols

The list covers the abbreviations and symbols used in this file. Symbols are reproduced as each source writes them, so the same quantity appears under more than one letter; the differences are notation rather than physical content.

Abbreviations used in this Supplementary Materials and in the main article.

Abbreviation	Meaning
6PPD	N-(1,3-dimethylbutyl)-N′-phenyl-*p*-phenylenediamine, an antidegradant
ACN	acrylonitrile
BR	butadiene rubber
CB	carbon black
CNF	crack-nucleation fatigue
DCB	double cantilever beam

Abbreviation	Meaning
DMA	dynamic mechanical analyser
EPDM	ethylene–propylene–diene rubber
FCG	fatigue crack growth
FE	finite element
FTF	fatigue to failure
HNBR	hydrogenated acrylonitrile–butadiene rubber
NBR	acrylonitrile–butadiene rubber
NR	natural rubber
phr	parts per hundred parts of rubber, by mass
pphr	the same quantity, written as its source writes it
SBR	styrene–butadiene rubber
SEN	single-edge-notched
SENT	single-edge-notched tension
SIC	strain-induced crystallisation
ZSC	a grade designation used by its source, which does not expand it

Symbols used in this Supplementary Materials, with the quantity each denotes.

Symbol	Meaning
T	tearing energy, the energy released per unit increase in crack area (J m^{-2})
G	strain-energy release rate, written with this symbol by the sources that prefer it (J m^{-2})
ΔG	cyclic strain-energy release rate range, used in the double cantilever beam row (J m^{-2})
W, U	strain-energy density of the uncracked body, as written by each source (J m^{-3})
h, h_0	undeformed specimen height between the grips (m). Both letters denote that same height; the subscript is the source's notation, not a second quantity
k	the geometry factor of the single-edge-notched tension expression, defined by its source in terms of the applied stretch
T_s, T_f	the two additive terms of a multi-dataset decomposition, defined by its source
L	specimen length between the free ends, as written by its source (m)
e, l_0	the crack-length correction offset and the unstrained gauge height of one source's tearing-energy expression (m)
a, c	crack length, as written by each source (m). In the power-law expression $\mathrm{d}c/\mathrm{d}N = AG^a$ reproduced from one source, a is instead the exponent, following that source's notation
a_0, a_f	initial and terminal crack length in a life integration (m)
N	number of cycles
$\mathrm{d}c/\mathrm{d}N$, $\mathrm{d}a/\mathrm{d}N$	crack growth per cycle, with the crack-length symbol each source uses
R	load ratio. The quantity it is formed from — force, displacement, strain or tearing energy — differs between sources and is stated in the row concerned
A, b	prefactor and exponent of a fitted power law, in the units its source states
T_L, T_{UL}	tearing energy evaluated on the loading and the unloading branch
T_d	a model term in a multi-dataset decomposition, defined by its source
β_f	a fixed exponent in that same decomposition

Table S1. Measurement-layer characteristics of thirteen fatigue crack-growth studies among the literature reviewed here. These thirteen studies were selected because together they span the geometry, energy-convention, load-minimum, frequency and thermal-state contrasts used in Sections 4–9 of the manuscript. The set is not a prevalence sample and is not the complete set of studies cited in this review. Row membership does not establish comparability. Numerical comparison is permitted only for the controlled contrast identified in the final column, and across rows only under the two-stage checklist of the main text (Section 3.5, Table 3): matching endpoint; coefficients paired with a common energy definition; geometry, load minimum, rate and history, and thermal state either matched or bounded by a within-study contrast measured on the same base polymer; and data reduction matched by re-deriving rates from the raw crack records. The Wang [34] and Shangguan [35] papers contain a substantially overlapping dataset and are treated as one dataset family.

Source	Material and endpoint	Geometry / constraint	Driving-force definition	Load minimum / ratio	Frequency and waveform	Thermal / environmental state	Quantitative range or parameter	Cross-study use
Kaang et al. [32]	N351 CB 50 phr NR and SBR; pre-cracked $\mathrm{d}c/\mathrm{d}N$	Pure shear, 2 × 200 × 20 mm; ~30-mm edge cut	$G = Uh_0$; $\mathrm{d}c/\mathrm{d}N = AG^a$	Fully relaxing, minimum strain 0	1 or 10 Hz; waveform not reported by the source; dynamic strain up to 100%	40 or 100 °C	$G \approx$ 300–60,000 J m^{-2}; source Table 2 a: NR 2.08/2.26/2.13/2.17, SBR 3.78/4.21/3.97/3.84 for 40 °C–1 Hz / 40 °C–10 Hz / 100 °C–1 Hz / 100 °C–10 Hz. The article's NR narrative range, 2.08–2.13, conflicts with these cells. The exponent varies little relative to the NR–SBR difference (NR 2.08–2.26, SBR 3.78–4.21); the temperature effect the article reports at a given tearing energy—about tenfold for NR and about hundredfold for SBR between 40 and 100 °C—resides in the prefactor A, which the article does not tabulate.	Paired within-study NR/SBR contrast, with the backbone not isolated from formulation (main text Table 6), and frequency contrast; the temperature contrast is controlled in direction, with its magnitude only approximate because the prefactor carrying it is not tabulated. Both the tabulated exponents and the conflicting narrative range are reported here, and neither is transferable to other formulations.
Narynbek Ulu et al. [41]	Six N772 70 phr HNBR blends; pre-cracked $\mathrm{d}c/\mathrm{d}N$	Mini-pure shear; two 3-mm in-situ edge notches	$T = Wh$; W from uncracked nominal stress–stretch response	Displacement $R = 0$	24.5-Hz stabilised FCG; sinusoidal; 2.5-Hz accommodation and energetic characterisation; preliminary tests at 2.5, 10.0 and 24.5 Hz showed no significant effect of frequency on the measured crack growth rate	DMA chamber set point 120 °C; specimen temperature not measured for the FCG tests	T = 50–1000 J m^{-2}; ≥6 rate measurements per target tearing-energy level and blend	Direct within-study ACN/hydrogenation contrast; no absolute plot rates before digitisation

Source	Material and endpoint	Geometry / constraint	Driving-force definition	Load minimum / ratio	Frequency and waveform	Thermal / environmental state	Quantitative range or parameter	Cross-study use
Soma et al. [33]	55 Shore A CB-filled NR; pre-cracked $\mathrm{d}c/\mathrm{d}N$ after thermal ageing	Pure shear, rubber section 2 × 200 × 20 mm; ~30-mm edge cut	$T = Uh$	Fully relaxing; displacement-controlled	3 Hz; waveform not reported by the source; tensile strain 25–65%	FCG at room temperature; prior air ageing at 70/100 °C for 24/72/120/168 h	$T \approx 10^{3.4}$–$10^{4.1}$ J m⁻²; power-law slope 1.77–2.01	Within-study ageing contrast only; ageing temperature must not be relabelled as FCG test temperature
Rublon and Favier [46]	N330-filled SBR with 6PPD 2 phr or 0 phr; pre-cracked $\mathrm{d}c/\mathrm{d}N$	Pure shear, 40 × 4 × 0.8 mm; two 5-mm edge notches	T from pure-shear strain-energy density and specimen height	Minimum/ratio not reported	5 Hz; waveform not reported; 300 cycles at 50% preconditioning; 5–50% energy characterisation	FCG at room temperature, 80 °C, or 100 °C; separate air ageing at 80 °C for 16/168/840 h	At 80 °C and 100 J m⁻², one specimen per formulation gives right/left rates of 0.00178/0.0128 nm/cycle (2-phr 6PPD) and 0.65/0.18 nm/cycle (0-phr); each low-energy material test lasted ~10 days at 80 °C, that is about 4 × 10⁶ cycles at 5 Hz, implying a total crack advance over the whole test of about 0.008 and 0.06 mm at the two 2-phr tips and about 2.8 and 0.8 mm at the two 0-phr tips; crack advance was read at paused intervals with a binocular microscope	Exploratory low-energy between-formulation contrast with two correlated crack tips per specimen and concurrent long thermal exposure; not an independent antioxidant effect size
Muñoz et al. [40]	50 phr silica-filled NR; instantaneous crack velocity and morphology. This is the only row whose endpoint is a time-based crack velocity; the other twelve rows report a per-cycle rate.	Pure shear, h = 20 mm, L = 220 mm; 30-mm edge precut	$G = Wh$; cycle-resolved force–displacement energy	Force returns to zero at cycle minimum; trimodal displacement control	1 Hz	Room temperature	No tabulated universal G–rate coefficient; high-G response alternates between two velocity states	Mechanism/morphology evidence; not a source for pooled power-law coefficients or a universal instability threshold
Shangguan et al. [35] / Wang et al. [34]	Isolator/filled-NR material; pre-cracked $\mathrm{d}a/\mathrm{d}N$ and dumbbell-life transfer	Edge-notched pure shear, 140 × 10 × 2 mm; 25-mm notch	$T = Wh_0$; W from unloading stress–strain curve; global crack-extension–cycle fit then differentiation	$R = T_{\min}/T_{\max} = 0$	Monotonically increasing variable-amplitude sinusoid; peak strain 0→0.75; 4 Hz	About 23 °C / room-temperature open air	For maximum tearing energy <2100 J m⁻²: $\mathrm{d}a/\mathrm{d}N$ [mm cycle⁻¹] = $4.65802 \times 10^{-12}\,(T_{\max}\,[\mathrm{J\,m^{-2}}])^{2.33362}$, r^2 = 0.9678; three physical specimens	Count once; protocol- and unit-specific data-reduction case. Derived rate points are not independent replicates and the fit is not a universal Paris law

Source	Material and endpoint	Geometry / constraint	Driving-force definition	Load minimum / ratio	Frequency and waveform	Thermal / environmental state	Quantitative range or parameter	Cross-study use
Tunnicliffe [36]	70 Shore A NR with 12 CB grades; pre-cracked $\mathrm{d}c/\mathrm{d}N$	Plane-strain pure-shear FCG	Separate loading T_{L} and unloading T_{UL} force–displacement definitions; T is obtained from a crack-length-corrected expression that compensates for the reduction in specimen cross-section as the crack extends and for the non-zero strain energy of the material in the cracked part of the specimen, with the correction constant $e = 0.28\, l_0$	Fully relaxing strain condition, $R = 0$	2.5 Hz; sinusoidal; 10,000-cycle preconditioning; ~15–50% peak strain	Room temperature	Source Table VII: b = 1.54–3.32 (T_{UL}) and 1.51–3.25 (T_{L}); separate $\log(A)$ by branch	Within-study CB contrast; never pool loading and unloading branches
Béranger et al. [97]	NBR/HNBR/HNBR-ZSC; pre-cracked $\mathrm{d}c/\mathrm{d}N$	Notched pure shear	$G = Wh$; the source does not identify the energy branch	Load ratio not reported	Frequency and waveform not reported	23 or 120 °C; separate 80 °C air ageing for 0/7/21 days	Quantitative 2500-nm/cycle versus 10-nm/min text pair rejected because the unit conflicts with the figure axis	Qualitative temperature direction and specified within-study formulation contrast only
El Maanaoui and Meier [42]	CB-filled EPDM and NR; FCG plus fatigue-to-failure	Ten simultaneous SENT specimens for FCG, thickness about 1.5 mm with a 1-mm single edge notch; axisymmetric dumbbell for FTF	SENT $T = 2\pi k W_{\mathrm{tot}} c$, where W_{tot} is the total energy density and therefore includes the hysteresis contribution, so it is systematically larger than the elastic strain-energy density used for T in the pure-shear rows; temperature-dependent Paris prefactor	FCG ratio not reported by the source	FCG loading applied as 50-ms pulses at a 10-Hz repetition frequency; FTF sinusoidal at 1 Hz; the waveform function within an FCG pulse is not reported	Chamber at 23/60/90 °C	Single exponent plus temperature-dependent prefactor reported; reusable numerical coefficient not tabulated in the article as published	Qualitative within-study temperature contrast; do not merge with pure-shear coefficients

Source	Material and endpoint	Geometry / constraint	Driving-force definition	Load minimum / ratio	Frequency and waveform	Thermal / environmental state	Quantitative range or parameter	Cross-study use
South et al. [26]	CB 65 phr NR; pre-cracked $\mathrm{d}a/\mathrm{d}N$	Modified DCB plane strain, in which a 2-mm rubber layer is bonded and cured between rigid metal adherends of a 266.7 × 25.4 mm beam, versus SEN plane stress	DCB ΔG evaluated by the modified beam theory method, which accounts for rotation at the delamination front, versus SEN Rivlin–Thomas tearing energy	$R = 0$, in load control for both the DCB and the SEN tests	DCB 2 Hz; SEN 0.25 Hz; waveform not reported	Room temperature	DCB rate reported about two orders faster than SEN on the compared energy scale	Bundled measurement-state contrast only; geometry, constraint, frequency, and energy convention change together
Hassim et al. [56]	NR/BR 70/30 with 0 or 10 pphr recycled tyre powder; stable $\mathrm{d}c/\mathrm{d}N$	SENT; initial crack 0.65 ± 0.15 mm	T from referenced energetic method; absolute/relative hysteresis densities also reported	Minimum load target 1 N; not equivalent to exact $R = 0$; the applied strain was displacement-controlled and compensated for permanent set throughout the FCG experiments	Sinusoidal, 5 Hz; displacement controlled; 40/59/71/96% dynamic strain	Ambient	Numerical T values require plot extraction	Conditional formulation comparison within strain level; the primary bar to combining these values with the pure-shear rows is the SENT geometry, in which T depends on crack length, rather than the load minimum
Kocjan et al. [52]	Non-crystallising rubber; FCG-to-CNF bridge	Planar-tension FCG, uniaxial CNF, and 3D dumbbell CNF	Geometry-specific $T(a)$ and integration from a_0 to a_f; thermal–FE transformation	Fully relaxing for stated uniaxial scope	Frequency varies with load/equipment limits	Room-temperature FCG reference; isothermal conversion for 3D dumbbell	The source gives about 20 h of testing for the crack-growth route against about 1600 h for the crack-nucleation route as its own serial-testing estimate; the schedule on which the tests were actually run is not stated	Bridge study rather than directly pooled FCG dataset; a_0, actual temperature, and frequency correction are required
Duncan et al. [21]	Filled/unfilled non-strain-crystallising rubbers; multi-dataset model	Pure-shear crack growth and trouser tear across datasets	$T = T_s + T_f$; four-variable continuous model with T_d	Dataset dependent / not reported	Method and dataset dependent	Room and elevated temperatures across source datasets	β_f = 4/3 fixed for examined rubbers; compound-specific remaining parameters	Model-synthesis axis; analyse each constituent dataset separately before comparison

Table notes

1. Natural rubber is abbreviated NR; missing metadata are written as “not reported” and never abbreviated NR.
2. Dimensions retain the order and labels reported by each source; they are not assumed to be mutually equivalent specimen dimensions. The same fidelity applies to symbols. Several pure-shear rows write what is the same relation as $G = Uh_0, T = Wh, T = Uh, G = Wh$ or $T = Wh_0$; the differences are source notation; the energy branch and conditioning state are stated in the row where the source reports them, and a row that does not state them leaves that item unresolved (main text Section 4.1).

3. T is the tearing energy in the Rivlin–Thomas sense, the energy released per unit increase in crack area; G is the strain-energy release rate, written with that symbol by the sources that prefer it; ΔG is the cyclic strain-energy release rate range reported in the double-cantilever-beam row. Symbol differences are source notation, and any substantive difference is stated in the row concerned. T, G, and ΔG are not pooled simply because their units are J m^{-2}: geometry, constraint, energy branch, and crack-length dependence remain part of the variable definition. The pure-shear rows also differ from one another in the ratio of initial crack length to specimen height, and this table does not assume that the pure-shear idealisation has been verified in every row.
4. The Béranger text pair at G = 100 J m^{-2} is excluded from all quantitative synthesis because nm/min conflicts with the plotted nm/cycle axis and the plotted locations do not support using it as an exact pair.
5. Wang [34] and Shangguan [35] share specimen values, fit coefficients, and life-validation structure. They are bibliographically retained but statistically counted once.
6. Each controlled contrast, rather than each paper or table row, is the unit of comparison. Composite rows are retained to expose confounding and bridge assumptions, not to imply internal statistical equivalence.
7. The driving-force column holds three kinds of entry: geometry-based measurement conventions, model-based decompositions, and life-integration schemes. The Duncan row inherits the conventions of the digitised source datasets it re-analyses rather than defining its own.
8. At a zero minimum and without residual deformation, the force, displacement and energy ratios coincide at zero. At a positive minimum they differ even without permanent set, because tearing energy is non-linear in deformation (main text Section 5.1), and permanent set separates them further, including at a nominal zero minimum.

Table S2. Prioritised experiments and data practices needed to convert condition-specific rubber fatigue-crack-growth evidence into transferable component predictions. Priorities are ordered by their ability to resolve a specific measurement or transfer ambiguity identified in this review. The proposed designs preserve endpoint separation and require direct observation of thermal, crack, and defect states rather than inferring them from machine settings alone. The second part of the table specifies Priorities 1–3, the three experiments proposed in Section 10 of the main article, by response variables, replication, crack resolution and comparison strategy.

Priority	Evidence gap	Proposed design	Minimum controls and outputs	Decision enabled	Evidence boundary
1	Geometry, constraint and energy-branch effects are usually inferred across different materials or protocols	Controlled round-robin on one fully documented compound using pure shear, SENT and DCB, with laboratories applying a pre-agreed energy-accounting convention	Common material batch and cure; matched R, waveform, temperature and crack observation; raw force–displacement and c–N; loading/unloading branches; within- and between-laboratory uncertainty	Quantify measurement-layer offsets and determine whether a geometry conversion is defensible	Does not create a universal correction factor; repeat for other material classes
2	Temperature, load ratio and frequency are confounded, while self-heating is often unmeasured	Factorial temperature × physically defined R × frequency map for strain-crystallising and non-crystallising reference compounds	Chamber, surface and bulk thermometry; waveform/dwell; thermal steady-state criterion; matched maximum driver; replicate hierarchy and SIC observation where applicable	Separate intrinsic test-temperature direction, non-relaxing/SIC response and cyclic heating	A chamber set point cannot substitute for specimen temperature
3	In the literature reviewed here, which was not searched with ozone or fluid terms, few studies examined ozone, oxygen, water or swelling during dynamic crack growth, including swelling in mineral oil, so no cross-study direction, magnitude or mechanism can be fixed (main text Section 8.5), and environmental conditioning is frequently mixed with the temperature of the subsequent FCG test	Ageing, ozone, oxygen, water, oil or other fluid exposures followed by an identical pre-cracked FCG protocol and an unexposed control	Exposure dose/time and mass/volume change; network/chemistry state; common post-exposure equilibration; identical FCG geometry, R, waveform and measured specimen temperature	Attribute a change to material-state conditioning rather than to a changed test protocol	A combined ozone × elevated-temperature FCG protocol was not found in the literature reviewed here; because ozone was not a query term in the executed search set, this is a bounded observation about the reports read for this review, not proof of absence
4	Coupon laws are rarely exercised under track-representative multiaxial, variable-amplitude histories	Apply field-informed spectra and multiaxial constraint to instrumented coupons/subcomponents, with constant-amplitude baselines	Published load sequence; local deformation and temperature histories; crack-path/mode observation; spectrum order and dwell; damage-rule benchmark	Test whether a coupon calibration transfers to a realistic duty cycle and identify spectrum/mode corrections	Track loads and wear/impact remain contextual until a validated bridge is demonstrated
5	Initial-defect size is commonly assumed and can be a major sensitivity in defect-controlled integrations	Measure process-dependent defect populations and propagate them through geometry-specific crack-growth integration	3D/2D defect metrology with detection limit; origin classification; a_0 distribution; stated a_f; parameter covariance; calibration and independent component validation	Produce probabilistic life and inspection-sensitive reliability estimates	Do not tune a_0 solely to recover observed life
6	Wear, cut-and-chip, initiation and pre-cracked FCG are described with overlapping fatigue language	Common observational programme that applies endpoint-specific tests to matched compounds and maps damage morphology without forcing one common coefficient	Synchronised force, temperature, imaging and surface/topography data; explicit endpoint criteria; transition/repeatability analysis	Establish when an FCG description is applicable and when contact/impact endpoints require a separate model	Endpoint bridges are hypotheses until validated; no direct pooling of rankings

Priority	Evidence gap	Proposed design	Minimum controls and outputs	Decision enabled	Evidence boundary
7	Published figures often prevent reanalysis of crack-rate extraction and energy accounting	Release the primary experimental datasets — raw crack-length–cycle, force–displacement, temperature and specimen-metadata files — with analysis code and provenance	Machine-readable units/schema; raw and processed levels; rejected intervals; versioned scripts; licences and persistent identifiers	Recompute rates under alternative admissible reduction methods and detect dataset-family reuse	Open data improve auditability but do not correct an under-specified experiment

Experimental specification for Priorities 1–3. Where neither the main article nor its sources fix a numerical minimum, the requirement is stated as a reporting requirement.

Specification	Priority 1: geometry round-robin	Priority 2: temperature × load ratio × frequency map	Priority 3: environmental conditioning
Principal response variables	$\mathrm{d}c/\mathrm{d}N$ against T for each geometry, with energy branch and fitted window stated; fitted C and β with their covariance; non-detection level and cycle count where the low-energy region is tested	$\mathrm{d}c/\mathrm{d}N$ at matched maximum driver in each cell; chamber, surface and bulk specimen temperature at thermal steady state; hysteresis loss per cycle; SIC observation where applicable	$\mathrm{d}c/\mathrm{d}N$–T curves of exposed and unexposed specimens under one protocol; exposure dose or time; mass or volume change; network or chemistry state after exposure
Replication hierarchy and recommended minimum	Specimens within laboratories within geometries, on one compound batch. At least one geometry repeated in more than one laboratory, so that between-laboratory variation can be separated from within-laboratory variation; more than one independent specimen per geometry and laboratory	More than one independent specimen per factorial cell; at least one strain-crystallising and one non-crystallising reference compound, with statements about a polymer class limited to the compounds tested	Exposed and control specimens from one batch and cure; more than one independent specimen per exposure level; exposure series and control tested in the same laboratory
Crack-resolution requirement	Crack-length resolution finer than the crack extension accumulated over one reading interval, reported with the interval, observation method and calibration (main text Section 4.6)	As Priority 1; in low-rate cells, the cycle count over which non-detection was established is also reported	As Priority 1; crack length and specimen dimensions measured in a stated reference state, and any swelling correction to the driving energy reported (main text Section 8.5)
Comparison strategy	Fitted coefficients compared with uncertainty intervals over the driving-force window common to all geometries; within-laboratory repeatability and between-laboratory reproducibility estimated separately on the repeated geometry; crack fronts, images and fitted points treated as subsamples of their specimen (main text Section 5.5)	Factor effects and interactions estimated with the specimen as the independent unit and the measured specimen temperature as a covariate, so that a frequency effect carried by self-heating is separated from a direct rate effect	Exposed–control differences in rate, with uncertainty intervals, over a common driving-force window, each exposure level compared with the control tested under the same protocol

Prioritisation logic

The order is a dependency chain rather than a ranking of importance: comparability has to be established before a transfer claim can be tested, and both have to be established before endpoint validity and reanalysability can be judged on a common basis. Each priority therefore presupposes the ones above it.

1. Priorities 1–3 resolve experimentally addressable comparability ambiguities before new constitutive complexity is added.
2. Priorities 4–5 address coupon-to-component transfer and potentially important defect/load uncertainties.
3. Priorities 6–7 protect endpoint validity and make future evidence re-analysable.
4. The agenda is intentionally bounded to the evidence gaps revealed by this review; it does not claim that these are the only important questions in elastomer fatigue.

Appendix S1

Appendix S1. Archived query record, update searches and targeted-retrieval stream for the literature underlying this review. This appendix documents how literature was discovered and retrieved: the twenty-six archived open-discovery queries of 20 July 2026 with their exact strings and yields, the screening outcome of that search, the full re-executions of 18 August and 24 September 2026, and the targeted retrieval that did not originate in the archived search. It records what was retrieved, not what was judged admissible. Which reports we read in full, which we judged eligible, and which we chose to cite rest on the appraisal described in Section 2 of the manuscript rather than on search arithmetic. Section 2 reports a failure of the first search attempt and how we diagnosed it, and Section 3 lists the retained search records and the terms on which they are available.

---

1. Archived open-discovery queries

We searched the Semantic Scholar Academic Graph on 20 July 2026, using the bulk paper-search endpoint of its public interface. We applied no lower publication-date restriction and no language filter. Each query was sent exactly as shown below. All fields were requested in a single API call per query, and each response occupied one API page on the execution date. The address we searched was https://api.semanticscholar.org/graph/v1/paper/search/bulk, and each search was sent as a single GET request carrying the query string itself in query and the list of record fields to return in fields. For every hit we asked for the paper identifier, title, abstract, year, authors, external identifiers, venue, publication types, publication date, journal, citation count, reference count, open-access location, URL and open-access status — fifteen fields, sent as the exact value paperId,title,abstract,year,authors,externalIds,venue, publicationTypes,publicationDate,journal,citationCount,referenceCount,openAccessPdf,url, isOpenAccess. We searched on 20 July 2026.

1.1 Result-set size, ordering, continuation and request spacing

We record these four parameters because they bear on a re-execution whose result sets are larger than the archived ones, although on the execution date none of them affected any count. They describe the retrieval behaviour that governs the counts reported here and are not a complete specification of the client.

- **limit**: not sent. The bulk endpoint's server-side page size was accepted as issued. The largest API-reported total across the twenty-six queries was 199 (OS5A), so every query was satisfied within one page and no result set approached the endpoint's bulk page capacity.
- **sort**: not sent. Records were consumed in the order the endpoint returned them. No count in this appendix depends on ordering, because every query was exported in full rather than truncated to a top-*n* slice.
- **Continuation token**: requested and handled. Where a search returns more records than fit in one response, the interface issues a token for the remainder, and we collected the remainder until no token was returned. On 20 July 2026 no search returned a token, which is why the Pages column below is 1 throughout. A search repeated today may exceed one response, and anyone repeating it should collect the remainder in the same way. Where a token was returned it was appended to the same query as token=<value>, and further pages of a query are archived as <QueryID>_page002.json and onward.
- **Spacing between queries**: We left eight seconds between successive searches, and the same interval between parts of a single search.

The twenty-six archived open-discovery queries, showing the concept block each belongs to, the exact string sent, and the yield recorded for it.

Query ID	Concept block	Exact query string	API-reported total	Exported rows	Pages
OS1A_CORE_RUBBER_FCG	Direct FCG	rubber AND "fatigue crack growth"	190	190	1
OS1B_CORE_ELASTOMER_FCG	Direct FCG	elastomer AND "fatigue crack growth"	56	56	1
OS1C_CORE_RUBBER_PROP	Direct FCG	rubber AND "fatigue crack propagation"	104	104	1
OS1D_CORE_ELASTOMER_PROP	Direct FCG	elastomer AND "fatigue crack propagation"	21	21	1
OS1E_CORE_RUBBER_CYCLIC	Direct FCG	rubber AND "cyclic crack growth"	5	5	1

Query ID	Concept block	Exact query string	API-reported total	Exported rows	Pages
OS1F_CORE_ELASTOMER_CYCLIC	Direct FCG	elastomer AND "cyclic crack growth"	5	5	1
OS1G_CORE_TEARING	Direct FCG	rubber AND "tearing fatigue"	12	12	1
OS1H_CORE_TEARING_ENERGY	Direct FCG	rubber AND fatigue AND "tearing energy"	113	113	1
OS2A_MATERIAL_SILICA	Material architecture	rubber AND "fatigue crack growth" AND silica	13	13	1
OS2B_MATERIAL_CB	Material architecture	rubber AND "fatigue crack growth" AND "carbon black"	39	39	1
OS2C_MATERIAL_NETWORK	Material architecture	rubber AND "fatigue crack growth" AND crosslink	11	11	1
OS2D_MATERIAL_SIC	Material architecture	rubber AND "fatigue crack growth" AND crystallization	34	34	1
OS3A_ENV_TEMPERATURE	Loading and environment	rubber AND "fatigue crack growth" AND temperature	36	36	1
OS3B_ENV_AGEING	Loading and environment	rubber AND "fatigue crack growth" AND aging	13	13	1
OS3C_ENV_HEATING	Loading and environment	rubber AND "fatigue crack growth" AND "self-heating"	2	2	1
OS3D_ENV_FREQUENCY	Loading and environment	rubber AND "fatigue crack growth" AND frequency	16	16	1
OS3E_ENV_VARIABLE	Loading and environment	rubber AND "fatigue crack growth" AND "variable amplitude"	5	5	1
OS4A_TRANSFER_LIFE	Component transfer	rubber AND "fatigue crack growth" AND "fatigue life"	24	24	1
OS4B_TRANSFER_COMPONENT	Component transfer	rubber AND "tearing energy" AND component	33	33	1
OS4C_TRANSFER_FE	Component transfer	rubber AND "fatigue crack growth" AND "finite element"	17	17	1
OS4D_TRANSFER_MULTIAXIAL	Component transfer	rubber AND fatigue AND multiaxial	105	105	1
OS4E_TRANSFER_TRACK	Component transfer	rubber AND fatigue AND track	155	155	1
OS5A_REVIEW_RUBBER	Prior reviews	rubber AND fatigue AND review	199	199	1
OS5B_REVIEW_ELASTOMER	Prior reviews	elastomer AND fatigue AND review	79	79	1
OS5C_REVIEW_FCG	Prior reviews	rubber AND "fatigue crack growth" AND review	8	8	1

Query ID	Concept block	Exact query string	API-reported total	Exported rows	Pages
OS5D_REVIEW_PROGRESS	Prior reviews	rubber AND "fatigue life" AND "research progress"	1	1	1

The archive logs the run rather than each query: twenty-five queries are stamped 2026-07-20T07:22:12Z and OS5D, which we added and ran separately, 2026-07-20T07:32:24Z. These stamps identify the logging run, not the moment an individual query was sent.

The twenty-six query exports contained 1,296 rows with overlap. Consolidating duplicates across queries by Semantic Scholar paper identifier left 864 unique records and removed 432 rows that repeated a record already returned by another query. These counts are historical results of the archived execution and may differ if the same queries are rerun after database updates.

The key by which we consolidated duplicates, and by which we later decided novelty in Section 4, is the Semantic Scholar paper identifier. That identifier is assigned by the database and is not guaranteed to be permanent: records can be merged, split or reissued between database states. The counts given here therefore describe the identifier state of the database on the dates stated, and a re-execution meeting a different identifier state can reproduce the queries exactly and still arrive at a slightly different unique-record total.

1.2 From the query exports into screening

Records passing from the archived queries into title-and-abstract screening, and the outcome of that screening.

Stage	Records
Unique records after consolidating duplicates across queries	864
Screened on title and abstract; forty records that matched a report we already held, by exact DOI or by normalised title, were handled through that existing record instead	824
Title-and-abstract outcome: excluded 420, retained for full text 389, left unresolved 15	824
Reports carried forward for full-text or metadata follow-up (389 retained plus 15 unresolved)	404

Thirty-eight of the 824 screened records matched a report we already held closely enough to raise a possible title collision but not closely enough to link with confidence; we kept them inside the screened set rather than setting them aside on the strength of an uncertain match.

The controlled reasons behind the 420 title-and-abstract exclusions, record by record, are held in the screening file listed in Section 3.

The follow-up list is where the record kept in this appendix ends. What became of those 404 reports — full-text retrieval, eligibility, and the decision to cite — rests on the judgements described in Section 2 of the manuscript rather than on search arithmetic, and the reference list of the review is its record.

2. Query-translation verification and re-execution

Before accepting any count from this search we checked whether the protocol's Boolean logic had survived translation into the API's query syntax. It had not. A first attempt on 20 July 2026 sent five long, nested Boolean strings, one per concept block, and every one of the five returned zero records, while a simple sentinel query sent to the same endpoint returned 190. Five nested strings returning nothing against a sentinel returning 190 identifies a failure of query translation and not an empty literature. We therefore discarded that attempt, re-issued each concept block as a set of short queries logged independently of one another, and re-executed the search. No record or count from the discarded attempt enters the screening flow or any number in this appendix. Its raw responses, its log and a written statement of the reason are retained unchanged in the authors' archive. The discarded attempt's hit export is a three-byte file containing only a byte-order mark, which is itself evidence that nothing was retrieved from it.

Re-issuing the concept blocks as short queries reduced the search vocabulary, and we record that reduction rather than describe the change as one of syntax alone. Each of the five nested strings had listed alternatives inside every concept, and the executed queries carry one term for each concept, so the alternatives were never sent. In the material block, rubber and elastomer stood alongside "natural rubber", "synthetic rubber" and the polymer abbreviations

EPDM, HNBR, NBR and SBR. In the crack-growth block they stood alongside "fatigue crack extension", "cyclic crack propagation", "crack growth rate" and "energy release rate". In the material-architecture block, silica, "carbon black" and crosslink stood alongside filler, network, nanocomposite, morphology and blend, and "strain induced crystallization" was narrowed to crystallization. In the loading-and-environment block, temperature, aging, "self-heating" and frequency stood alongside thermal, oxidation, ozone, waveform, dwell, fluid, oil and water. In the component-transfer block, component, "finite element", multiaxial and track stood alongside durability, "initial flaw", defect, spectrum, tyre, tire, isolator and mount. In the prior-reviews block, review stood alongside fracture, overview and "state of the art". The nested strings had also carried spelling variants of terms that were themselves retained, ageing beside aging and "self heating" beside "self-heating", and the endpoint treats those as distinct tokens. Section 6 states what follows for statements of absence.

Both attempts wrote a query-definition file of the same name, frozen_queries.json, and the archive holds both, with different contents and different SHA-256 values. The entry in Section 3 is the file from the executed run and is identified as such.

3. Archive-integrity identifiers

The following SHA-256 values identify the files from which we prepared this appendix. They are given so that any copy supplied on request can be checked against the record used here. The last column states what can be obtained for each file.

The archived files behind this appendix, with their format, SHA-256 value and availability.

Archived file	Format	SHA-256	Availability
frozen_queries.json — query definitions of the executed run of 20 July 2026 (Section 1)	JSON	CBA28AFA604749E271210FAB0EAD1FCD EBFABD111CC890ECC4C9EE2AD4141391	On request
semantic_scholar_search_log.csv — query log of that run	CSV	B3427D7AE887B7A61BCF104880AA30D86 D1B4487868DBBA1660AEDA2C35FA597	On request
semantic_scholar_all_query_hits.csv — full query export, carrying abstract text	CSV	EFDEBABBBA20033F4C366BB6B8F9BFFF 1C2C3044A0DF1B1CBFA9ACDE4A5CA825	Not distributed

Archived file	Format	SHA-256	Availability
semantic_scholar_query_hits_metadata_no_abstract.csv — the export available on request in its place	CSV	31FA00A68935D48E679B44A43CD05B08B9A8B060F8A65B84AF4F550F6AAD137E	On request
screening_disposition_no_abstract.csv — title-and-abstract decisions for the 824 screened records (Section 1.2)	CSV	A980B439E097AFADE58BE2ADEAEDA4743363179BCB85F4D20888EC12DE314149	On request
delta_search_log.csv — query log of the re-execution of 18 August 2026 (Section 4)	CSV	0606060E29CDDDFAFBEEE21F1C57646F6C303C0B475B0A916542859F86BEBFD1	On request
delta_search_summary.json — run summary of that re-execution, including its two-and-a-half-minute duration	JSON	23884D3A7958EC33B585C767D0AD46DBC0F4FEFF7817FE7F5A10E9ECCE7B1DD2	On request
delta_search_log.csv — query log of the re-execution of 24 September 2026 (Section 4)	CSV	EEDA89A6BB69849BB0BCEC61DF35917BBB984C7EBA093B3AAD8713770BFB19FA	On request
delta_search_summary.json — run summary of the re-execution of 24 September 2026	JSON	93AF1A5EA1ECBDBC5AC07165AB96FBBA53CA1EC9D7A8D7476E593822B00E236B	On request
Written statement of the discarded first attempt (Section 2)	Markdown	3859B3EB0B544404907610F2E567C777EEFACEA09FF1A58B6C4035E385FE8255	On request
CHASE_CANDIDATES.md — citation-chasing candidate list of 19 August 2026 (Section 5.2)	Markdown	EADABB92DA19FEA15F080AD5F59A71C2285A1B46B20754DC5F2AF30323966838	On request
ACQUISITION_MANIFEST.csv — full-text acquisition manifest for 18–19 August 2026 (Section 5.2)	CSV	3C56FAE19913534E65F5728DF606A06507DDBB84B665B6D0F1896D65E09DA9FE	On request

We do not distribute the export semantic_scholar_all_query_hits.csv, because it carries the abstract text returned by the API. The file available on request in its place is not that file with one column blanked. The withheld export has eighteen columns and the supplied export has sixteen: abstract was removed for the reason just given, and open_access_pdf was removed because it holds third-party file locations whose availability and licence status change over time and which we cannot warrant. Every remaining column is carried over unchanged, and consolidating duplicates in the supplied export by paper identifier reproduces the same 1,296 rows and 864 unique records reported in Section 1. A reader who needs an abstract or an open-access link for a listed record must obtain it from the database rather than from this export. The checksum of the withheld file is listed for completeness of the record; it is not a value a reader can check against a file they receive.

4. Update searches, 18 August and 24 September 2026

We re-executed the twenty-six archived queries in full on 18 August 2026 and again on 24 September 2026, when we closed the search. The August re-run returned 1,302 records and

868 unique paper identifiers, against 1,296 and 864 on 20 July 2026. One response per query is retained in the archive.

We determined novelty by a paper-identifier and DOI difference against the July archive rather than by a publication-date filter, because a date filter excludes records published before the cut-off and indexed after it — exactly the records an update search exists to catch. That choice changed the outcome: of the four records new in August, one had been published on 3 April 2026 and would have been invisible to a date-filtered query.

The comparison is at the level of identifiers and DOIs only. We narrowed the requested field list for the re-run to title,year,publicationDate,externalIds,venue,publicationTypes,abstract — seven fields against the fifteen requested in July — because the re-run was a difference check rather than a fresh retrieval. A record-by-record comparison of the two exports is therefore not possible and none is claimed: what the re-run establishes is which identifiers and DOIs were present in August and absent in July, not whether the metadata of the records common to both had changed in the interval. The query strings, the endpoint and the names of the two query parameters were those of Section 1; the interval we set between queries was shorter than in July — three and a half seconds rather than eight — and every query again returned its full reported total in a single response, so the difference in interval has no bearing on which records were returned.

Four records were new, all new by DOI as well as by identifier, and none of them appeared in the screened set of Section 1.2:

The four records returned by the re-execution of 18 August 2026 that the July archive did not contain, and what we did with each.

DOI	Published	Venue	Outcome
10.1002/adma.74087	2026-07-21	Advanced Materials	full text retrieved; retained [10]
10.1007/s11837-026-08273-x	2026-04-03	JOM	out of scope: endpoint is not pre-cracked FCG
10.3390/buildings16153131	2026-08-06	Buildings	out of scope: asphalt pavement

DOI	Published	Venue	Outcome
10.1002/adma.202522702	2026-08-05	Advanced Materials	out of scope: photonics

The venue column gives the journals' full titles. For the two *Advanced Materials* records the archived responses carry the database's own variant string, Advances in Materials, in the venue field; we have normalised it here, and a reader checking this table against the archived responses will meet the variant rather than the form printed above.

Twenty-nine days of the same queries therefore yielded one report that we took into the review. We took four further reports from the full-text stage of the principal search over the same period. The five we retained are 10.1002/adma.74087 [10], 10.1039/d6mh00677a [9], 10.1146/annurev-matsci-101922-122133 [11], 10.5254/rct.25.00032 [27] and 10.1016/j.engfracmech.2026.111875 [51].

The second re-execution, on 24 September 2026, used the same queries, endpoint, fields and procedure as that of 18 August. It returned 1,319 records and 877 unique paper identifiers. Nine identifiers and DOIs were absent from both earlier archives. One is within the scope of the review: a phenomenological model for fatigue crack growth in rubber published online on 11 September 2026 (10.5254/rct.26.00022). Its full text was not accessible to us when we closed the search, and because an abstract alone cannot support a statement in the review, we did not cite it; its abstract does not conflict with any statement the review makes. The other eight address asphalt, dentistry, wheel–rail creep, tyre abrasion, crack initiation in seals and the stress–life fatigue of bushings, none of which is a pre-cracked fatigue crack-growth endpoint, and we took none of them into the review.

5. Citation-based retrieval stream

Section 2 of the manuscript names targeted, citation-based retrieval as a route by which we brought literature into the review. This section gives its scope and dates.

5.1 Retrieval during writing (from July 2026)

Writing raised specific technical questions that the archived open search had not been built to answer — where the tearing-energy definition originates, how a threshold is derived, on what basis a specimen geometry is held to give crack-length independence — and for each such question we went to the primary report in which the point was established. We brought much of the classical fracture-mechanics literature into the review by this route. The archived open search did not return these reports, and we identify them as targeted retrievals rather than as its output.

5.2 Backward citation chasing, 19 August 2026

We ran a single, dated pass over the reference lists of the five reports retained in Section 4. Only two of the five, [10] and [27], print DOIs in their reference lists; [9], [11] and [51] do not, so the DOIs collected in this pass come from the reference lists of those two. It yielded 148 unique DOIs; nine were already cited, leaving 139 candidates, which we graded by subject scope against the review's inclusion criteria: sixteen in the rubber and elastomer FCG mainline, twenty-four in network and toughness, eighteen in hydrogels, and eighty-one outside the scope of the review.

We then applied a stated retrieval rule to those grades. We retrieved all sixteen of the FCG mainline. Of the twenty-four network and toughness candidates we retrieved the six that bear directly on the crosslink-density and network-topology argument of Section 7.4 of the manuscript, which is the point at which that literature is actually used. We retrieved none of the eighteen hydrogel candidates; hydrogels lie outside the scope of this review. We retrieved none of the eighty-one out-of-scope candidates. That rule accounts for the thirty-six in-scope candidates we did not retrieve as a judgement about marginal use rather than as an unexplained shortfall.

The rule yielded twenty-two reports, which we retrieved and read in full. Twenty-one were cited. The twenty-second proved to be the peer-reviewed journal version of a technical report already in the reference list, so we replaced that entry rather than duplicating it; the replaced entry is reference [22]. None of the twenty-two was cited without reading its full text. The bibliographic details of the twenty-one cited entries are taken from Crossref structured records rather than from the reference lists in which their DOIs were found.

Two limits bound this pass. First, text extraction failed on twenty-seven further entries in the two DOI-printing reference lists; these lie in the same candidate pool from which we were retrieving only the FCG mainline and the six network entries that attach to one specific argument, so what is at risk is a small number of further candidates in those two grades. Second, the reference lists of [9], [11] and [51], which print no DOIs, were not harvested. The candidate list of 19 August 2026 records a second pass by author–title reverse lookup as an option for both; we did not take it up, and citation chasing was a single round, not iterated to saturation.

- The candidate list and the acquisition manifest are listed in Section 3. The manifest covers every full text we obtained on 18–19 August 2026, thirty-five in all, and identifies each by DOI and records what we did with it. The twenty-two of this pass are one part of that total; the remainder are the five reports of Section 4 and eight further reports that we obtained and, on reading them, did not use.
- Four of the twenty-two are scanned page images with no text layer. We read those four from the scan and typed the values we quote from them, so their page markers are the original journal pages rather than PDF pages, and the point at which each such value is used is identified. No text from any source article is redistributed with this

review: this appendix and its tables report values, page locations and measurement conditions, not the wording of the sources.

5.3 Boundary

The reports in Section 5 are not products of the search in Section 1 and are not counted in its flow numbers. They are recorded as targeted retrievals, and are kept separate from the search of Section 1 so that the two populations cannot be conflated.

6. Scope of this appendix

This appendix documents discovery and retrieval. Inclusion in a query export did not confer eligibility, and nothing here presents the search as systematic or exhaustive. Full-text access, endpoint, evidence role, page locator, measurement state, dataset origin, and claim-level admissibility were assessed separately as described in Section 2 of the manuscript.

Two limits of the archived vocabulary follow from Section 2 and bear on how the review's negative statements should be read. The executed queries use one term for each concept, so terms such as ozone, oxidation, waveform, dwell and fluid exposure, and the polymer abbreviations, were never sent; and the queries were confined to rubber and elastomers throughout. Where the review reports that something was not found, the reference point of that statement is the literature reviewed here — the reports in its reference list — and not the reach of the archived queries. "Not found in the literature reviewed here" should be read as exactly that: a bounded observation about a named set of reports, and not as a claim that the archived search would have returned such a report had one existed.

Comprehensiveness is not what the argument of this review rests on. The argument is built from contrasts made within single studies and from the measurement state recorded alongside each result, so it turns on how well each result is conditioned rather than on how often a finding recurs across the literature. Coverage is accordingly not claimed here; it is recorded

here — the exact strings, the yields, the screening outcome, the re-execution, and the retrieval that lies outside the queries — in enough detail for a reader to judge what the search reached and what it did not.

Note on identifiers: this appendix contains no Supplementary Table. The OS1A–OS5D labels in Section 1 are query identifiers, not table numbers. Tables S1 and S2 are provided in the same supplementary file.

References cited in the Supplementary Materials

Reference numbers are those of the main article.

[9] Ning, D.; Yang, M.; Shi, Q. Polymer fatigue: mechanism, mechanics and design. *Mater. Horiz.* **2026**, *13*, 8434–8463. https://doi.org/10.1039/d6mh00677a

[10] Baur, E.; Amstad, E. Can elastomers combine stiffness, toughness and fatigue resistance? *Adv. Mater.* **2026**, e74087. https://doi.org/10.1002/adma.74087

[11] Steck, J.; Ahn, C.H.; Suo, Z. Polymers resist fatigue crack growth by deconcentrating stress. *Annu. Rev. Mater. Res.* **2025**, *55*, 333–358. https://doi.org/10.1146/annurev-matsci-101922-122133

[21] Duncan, A.M.; Akutagawa, K.; Papageorgiou, D.G.; Ramier, J.L.; Busfield, J.J.C. A Unified Equation for Predicting Crack Growth in Rubber Composites Across All Crack Growth Rates. *Polymers* **2025**, *17*, 1357. https://doi.org/10.3390/polym17101357

[22] Gent, A.N.; Tobias, R.H. Threshold tear strength of elastomers. *J. Polym. Sci. Polym. Phys. Ed.* **1982**, *20*, 2051–2058. https://doi.org/10.1002/pol.1982.180201107

[26] South, J.T.; Case, S.W.; Reifsnider, K.L. Crack growth of natural rubber using a modified double cantilever beam. *Mech. Mater.* **2002**, *34*, 451–458. https://doi.org/10.1016/S0167-6636(02)00173-4

[27] Yamabe, J.; Yamamoto, R.; Amino, N.; Kakubo, T.; Maeda, N.; Fujikawa, M.; Koishi, M. Fatigue crack growth behavior and mechanistic insights of carbon black- and silica-filled

styrene-butadiene rubbers. *Rubber Chem. Technol.* **2025**, *98*, 674–693. https://doi.org/10.5254/rct.25.00032

[32] Kaang, S.; Jin, Y.W.; Huh, Y.-I.; Lee, W.-J.; Im, W.B. A test method to measure fatigue crack growth rate of rubbery materials. *Polym. Test.* **2006**, *25*, 347–352. https://doi.org/10.1016/j.polymertesting.2005.12.005

[33] Soma, P.; Tada, N.; Uchida, M.; Nakahara, K.; Taga, Y. A Fracture Mechanics Approach for Evaluating the Effects of Heat Aging on Fatigue Crack Growth of Vulcanized Natural Rubber. *J. Solid Mech. Mater. Eng.* **2010**, *4*, 727–737. https://doi.org/10.1299/jmmp.4.727

[34] Wang, X.-L.; Liao, M.-Y.; Xu, Y.; Liu, X.-A. Fatigue crack propagation characteristics of rubbery materials under variable amplitude loading. *Results Phys.* **2018**, *10*, 233–240. https://doi.org/10.1016/j.rinp.2018.05.043

[35] Shangguan, W.; Wang, X.; Duan, X.; Liu, G.; Yan, J. Fatigue Crack Growth Experiment and Modeling for Rubbers Used in Isolators under Variable Amplitude Loads. *J. Mech. Eng.* **2015**, *51*, 50–58. (In Chinese) https://doi.org/10.3901/jme.2015.08.050

[36] Tunnicliffe, L.B. Fatigue crack growth behavior of carbon black-reinforced natural rubber. *Rubber Chem. Technol.* **2021**, *94*, 494–514. https://doi.org/10.5254/rct.21.79935

[40] Muñoz, L.; Vanel, L.; Sanseau, O.; Sotta, P.; Long, D.; Odoni, L.; Guy, L. Fatigue crack growth dynamics in filled natural rubber. *Plast. Rubber Compos.* **2012**, *41*, 273–276. https://doi.org/10.1179/1743289812y.0000000013

[41] Narynbek Ulu, K.; Huneau, B.; Verron, E.; Béranger, A.S.; Heuillet, P. Effects of acrylonitrile content and hydrogenation on fatigue behaviour of HNBR. *Fatigue Fract. Eng. Mater. Struct.* **2019**, *42*, 1578–1594. https://doi.org/10.1111/ffe.12974

[42] El Maanaoui, H.; Meier, J. Lifetime prediction with temperature dependence for EPDM and NR elastomers based on fatigue crack growth mechanical measurements and filler distribution. *Polymer* **2021**, *228*, 123909. https://doi.org/10.1016/j.polymer.2021.123909

[46] Rublon, P.; Favier, A. Effect of Antioxidants on the Fatigue Crack Growth Behavior of Filled SBR Compounds. *Procedia Eng.* **2015**, *133*, 161–170. https://doi.org/10.1016/j.proeng.2015.12.644

[51] Liu, W.; Xu, Y.; Liu, Y.; Zhang, C.; Zhang, Q.; Cui, J. A novel ring specimen for fatigue crack growth rate testing of rubber with full-cycle crack length monitoring. *Eng. Fract. Mech.* **2026**, *333*, 111875. https://doi.org/10.1016/j.engfracmech.2026.111875

[52] Kocjan, T.; Nagode, M.; Klemenc, J.; Oman, S. Coupling of fatigue crack growth and crack nucleation fatigue approach for non-crystallising rubber under fully relaxing uniaxial loading with multiaxial stress–strain state of 3D dumbbell test specimen. *Polym. Test.* **2025**, *145*, 108752. https://doi.org/10.1016/j.polymertesting.2025.108752

[56] Hassim, D.H.A.I.; Abraham, F.; Summerscales, J.; Brown, P. Fatigue Crack Growth of Natural Rubber/Butadiene Rubber Blend Containing Waste Tyre Rubber Powders. *Solid State Phenom.* **2021**, *317*, 293–299. https://doi.org/10.4028/www.scientific.net/ssp.317.293

[97] Béranger, A.S.; Qin, J.; Heuillet, P.; Baurier, H. Fatigue crack growth behavior of NBR, HNBR, HNBR ZSC compounds. *Procedia Eng.* **2018**, *213*, 145–152. https://doi.org/10.1016/j.proeng.2018.02.016
